# The importance of considering competing risks in recurrence analysis of intracranial meningioma

**DOI:** 10.1007/s11060-024-04572-y

**Published:** 2024-02-10

**Authors:** Christian Mirian, Lasse Rehné Jensen, Tareq A. Juratli, Andrea Daniela Maier, Sverre H. Torp, Helen A. Shih, Ramin A. Morshed, Jacob S. Young, Stephen T. Magill, Luca Bertero, Walter Stummer, Dorothee Cäcilia Spille, Benjamin Brokinkel, Soichi Oya, Satoru Miyawaki, Nobuhito Saito, Martin Proescholdt, Yasuhiro Kuroi, Konstantinos Gousias, Matthias Simon, Jennifer Moliterno, Ricardo Prat-Acin, Stéphane Goutagny, Vikram C. Prabhu, John T. Tsiang, Johannes Wach, Erdem Güresir, Junkoh Yamamoto, Young Zoon Kim, Joo Ho Lee, Matthew Koshy, Karthikeyan Perumal, Mustafa K. Baskaya, Donald M. Cannon, Dennis C. Shrieve, Chang-Ok Suh, Jong Hee Chang, Maria Kamenova, Sven Straumann, Jehuda Soleman, Ilker Y. Eyüpoglu, Tony Catalan, Austin Lui, Philip V. Theodosopoulos, Michael W. McDermott, Fang Wang, Fuyou Guo, Pedro Góes, Manoel Antonio de Paiva Neto, Aria Jamshidi, Ricardo Komotar, Michael Ivan, Evan Luther, Luis Souhami, Marie-Christine Guiot, Tamás Csonka, Toshiki Endo, Olivia Claire Barrett, Randy Jensen, Tejpal Gupta, Akash J. Patel, Tiemo J. Klisch, Jun Won Kim, Francesco Maiuri, Valeria Barresi, María Dolores Tabernero, Simon Skyrman, Anders Broechner, Mathias Jacobsen Bach, Ian Law, David Scheie, Bjarne Winther Kristensen, Tina Nørgaard Munch, Torstein Meling, Kåre Fugleholm, Paul Blanche, Tiit Mathiesen

**Affiliations:** 1 Department of Neurosurgery, Copenhagen University Hospital, Copenhagen, Denmark; 2 Department of Neurosurgery, Division of Neuro-Oncology, Faculty of Medicine and University Hospital Carl Gustav Carus, Technische Universität Dresden, 01307 Dresden, Germany; 3 Department of Neurosurgery, Laboratory of Translational Neuro-Oncology, Massachusetts General Hospital Cancer Center, Harvard Medical School, Boston, USA; 4 Department of Pathology, Bartholin Institute, Rigshospitalet, Copenhagen University Hospital , Copenhagen, Denmark; 5 Department of Clinical and Molecular Medicine, Faculty of Medicine and Health Sciences, Norwegian, University of Science and Technology (NTNU), Laboratory Centre, St. Olavs Hospital, NO-7491 Trondheim, Norway; 6 Department of Pathology, Laboratory Centre, St. Olavs Hospital, NO-7030 Trondheim, Norway; 7 Department of Radiation Oncology, Massachusetts General Hospital, Harvard Medical School, Boston, MA USA; 8 Department of Neurological Surgery, University of California San Francisco, San Francisco, CA USA; 9 Department of Neurological Surgery, Northwestern University, Feinberg School of Medicine, Illinois, USA; 10 Pathology Unit, Department of Medical Sciences, University and Città Della Salute E Della Scienza University Hospital of Turin, Turin, Italy; 11 Department of Neurosurgery, University of Münster, Münster, Germany; 12 Institute for Neuropathology, University of Münster, Münster, Germany; 13 Department of Neurosurgery, Saitama Medical Center/University, Saitama, Japan; 14 Department of Neurosurgery, The University of Tokyo Hospital, Tokyo, Japan; 15 Department of Neurosurgery, University Regensburg Medical Center, Regensburg, Germany; 16 Department of Neurosurgery, Adachi Medical Center, Tokyo Women’s Medical University, Tokyo, Japan; 17 Department of Neurosurgery, Athens Medical Center, Athens, Greece; 18 Department of Neurosurgery, Bethel Clinic University of Bielefeld Medical Center, Bielefeld, Germany; 19 Department of Neurosurgery, Yale School of Medicine Yale New Haven Hospital, Smilow Cancer Hospital, New Haven, USA; 20 Department of Neurosurgery, Hospital La Fe, Valencia, Spain; 21 Department of Neurosurgery, Université Paris Cité, Beaujon Hospital, Assistance Publique Hôpitaux de Paris, Paris, France; 22 Department of Neurological Surgery, Loyola University Medical Center, Stritch School of Medicine, Illinois, USA; 23 Department of Neurosurgery, University Hospital Leipzig, Leipzig, Germany; 24 Department of Neurosurgery, University of Occupational and Environmental Health, Kitakyushu, Japan; 25 Department of Neurosurgery, Samsung Changwon Hospital, Sungkyunkwan University School of Medicine, Changwon, Republic of Korea; 26 Department of Radiation Oncology, Seoul National University Hospital, Seoul National University College of Medicine, Seoul, Republic of Korea; 27 Department of Radiation Oncology, University of Illinois Hospital and Health Sciences System, Illinois, USA; 28 Department of Neurosurgery, University of Wisconsin School of Medicine and Public Health, Madison, WI USA; 29 Department of Radiation Oncology Spencer Fox Eccles School of Medicine, University of Utah, Salt Lake City, UT USA; 30 Department of Radiation Oncology, Yonsei University College of Medicine, Seoul, Republic of Korea; 31 Department of Neurosurgery, Yonsei University College of Medicine, Seoul, Republic of Korea; 32 Department of Neurosurgery, University Hospital Basel, Basel, Switzerland; 33 Division of Neurosurgery, Miami Neuroscience Institute, Miami, FL USA; 34 Department of Neurosurgery, The First Affiliated Hospital of Zhengzhou University, Zhengzhou, Henan China; 35 Department of Neurosurgery, Federal University of São Paulo, São Paulo, Brazil; 36 Department of Neurological Surgery, Sylvester Comprehensive Cancer Center, University of Miami, Miami, FL USA; 37 Division of Radiation Oncology, McGill University Health Centre, McGill University, Montreal, QC Canada; 38 Department of Pathology, McGill University Health Centre, Montreal, QC Canada; 39 Department of Pathology, Faculty of Medicine, University of Debrecen, Debrecen, Hungary; 40 Division of Neurosurgery, Tohoku Medical and Pharmaceutical University, Tohoku, Japan; 41 Infirmary Cancer Care, Montgomery, AL USA; 42 Department of Neurosurgery, Huntsman Cancer Institute, University of Utah, Salt Lake City, UT USA; 43 Department of Radiation Oncology ACTREC, Tata Memorial Centre, HBNI Kharghar, Navi Mumbai, 410210 India; 44 Department of Neurosurgery, Baylor College of Medicine, Houston, TX USA; 45 Department of Otolaryngology-Head and Neck Surgery, Baylor College of Medicine, Houston, TX USA; 46 Jan and Dan Duncan Neurological Research Institute, Texas Children’s Hospital, Houston, TX USA; 47 Department of Molecular and Human Genetics, Baylor College of Medicine, Houston, TX USA; 48 Department of Radiation Oncology, Gangnam Severance Hospital, Yonsei University College of Medicine, Seoul, Republic of Korea; 49 Department of Neurosurgery, University of Naples Federico II, Naples, Italy; 50 Department of Diagnostics and Public Health, University of Verona, Verona, Italy; 51 Instituto de Investigación Biomédica de Salamanca (IBSAL), University Hospital of Salamanca, Salamanca, Spain; 52 Department of Clinical Neuroscience, Karolinska Institutet, Stockholm, Sweden; 53 Department of Clinical Physiology and Nuclear Medicine, Copenhagen University Hospital-Rigshospitalet, Copenhagen, Denmark; 54 Department of Clinical Medicine, Faculty of Health and Medical Sciences, University of Copenhagen, Copenhagen, Denmark; 55 Department of Clinical Medicine and Biotech Research and Innovation Center (BRIC), University of Copenhagen, Copenhagen, Denmark; 56 Department of Epidemiology Research, Statens Serum Institut, Copenhagen, Denmark; 57 Department of Neurological Surgery, Istituto Nazionale Neurologico “C.Besta”, Milan, Italy; 58 Section of Biostatistics, Department of Public Health, University of Copenhagen, Copenhagen, Denmark

**Keywords:** Meningioma, Neuro-oncology, Competing risk, Recurrence

## Abstract

**Background:**

The risk of recurrence is overestimated by the Kaplan–Meier method when competing events, such as death without recurrence, are present. Such overestimation can be avoided by using the Aalen-Johansen method, which is a direct extension of Kaplan–Meier that accounts for competing events. Meningiomas commonly occur in older individuals and have slow-growing properties, thereby warranting competing risk analysis. The extent to which competing events are considered in meningioma literature is unknown, and the consequences of using incorrect methodologies in meningioma recurrence risk analysis have not been investigated.

**Methods:**

We surveyed articles indexed on PubMed since 2020 to assess the usage of competing risk analysis in recent meningioma literature. To compare recurrence risk estimates obtained through Kaplan–Meier and Aalen-Johansen methods, we applied our international database comprising ~ 8,000 patients with a primary meningioma collected from 42 institutions.

**Results:**

Of 513 articles, 169 were eligible for full-text screening. There were 6,537 eligible cases from our *PERNS* database. The discrepancy between the results obtained by Kaplan–Meier and Aalen-Johansen was negligible among low-grade lesions and younger individuals. The discrepancy increased substantially in the patient groups associated with higher rates of competing events (older patients with high-grade lesions).

**Conclusion:**

The importance of considering competing events in recurrence risk analysis is poorly recognized as only 6% of the studies we surveyed employed Aalen-Johansen analyses. Consequently, most of the previous literature has overestimated the risk of recurrence. The overestimation was negligible for studies involving low-grade lesions in younger individuals; however, overestimation might have been substantial for studies on high-grade lesions.

**Supplementary Information:**

The online version contains supplementary material available at 10.1007/s11060-024-04572-y.

## Introduction

The risk of tumor recurrence is a relevant endpoint in studies on meningioma patients. Statistical analyses are often performed using the Kaplan–Meier method, which was originally constructed to analyze the risk of death. In recurrence analysis, the Kaplan–Meier method is problematic because it considers a recurrence the only possible outcome and consequently produces biased risk estimates when competing risks are present. A competing risk is defined as an event that prevents the occurrence of the primary event of interest (in this case, recurrence). In studies on risk of meningioma recurrence, death before having a recurrence is a competing event since death precludes the possibility of ever experiencing the recurrence. Consequently, patients that are “recurrence-free” at a given time point could denote patients that either have died without a recurrence, or patients that are alive without having experienced a recurrence at that time yet. In the case where patients are censored as “recurrence-free”, but not distinguished as dead (and no longer at risk) *vs*. alive (and still at risk), the Kaplan–Meier method will lead to erroneous overestimation of the risk [1, 2]. The Kaplan–Meier method fails to acknowledge that patients who die without experiencing a recurrence are no longer at risk of recurrence. The only risk that can be estimated without bias using the Kaplan–Meier method in this context is the risk of recurrence in a hypothetical and irrelevant population of meningioma patients who cannot die until they have experienced a recurrence [3, 4].

The Aalen-Johansen method is a simple and direct extension of the Kaplan–Meier method that produces unbiased risk estimates in the presence of competing risks [5, 6]. It is, therefore, relevant to meningioma research, since most lesions occur in older individuals and display slow-growing properties, which implies that many patients die without ever experiencing a recurrence. From a statistical perspective, the Aalen-Johansen method provides correct estimates for recurrence risk analysis, whereas the Kaplan–Meier method yields biased results. The extent of the application of these methods in meningioma research, as well as the potential impact of biased estimates on published findings in existing literature, remains unclear.

The aims of this study are to (1) provide an overview of the typical methodology used for recurrence risk estimation in the recent meningioma literature, and to (2) compare risk estimates of recurrence obtained with the Aalen-Johansen method *vs*. the Kaplan–Meier method.

## Methods

A search was conducted on PubMed on February 17th, 2023, to identify studies relevant for the assessment of methodologies used in recent literature for estimating recurrence risk. The following search string was used: *Meningioma[MeSH] AND (recurrence[title/abstract] OR progression[title/abstract]*), and was restricted to papers published between 2020 and the search date. The methodology used to estimate recurrence risk (i.e. the Kaplan–Meier or the Aalen-Johansen method) was extracted from the included studies.

To compare risk estimates of recurrence between the Kaplan–Meier and Aalen-Johansen methods, we evaluated meningioma outcomes from the PERNS database (PERsonalized NeuroSurgery). This database includes data from ~ 8,000 meningioma patients from 42 centers spanning six continents registered locally by the treating or diagnostic physicians. Herein, all meningioma patients were included at the time of their primary disease, which was between 1991 and up until 2019. The database includes information about time from diagnosis to recurrence and death, World Health Organization (WHO) grading (2016, 2007, Older), Simpson grade, radiotherapy (and total Gy dosage if applied), the Ki-67 proliferation index, age at diagnosis, sex, tumor location, and treating center.

**Fig. 1:**
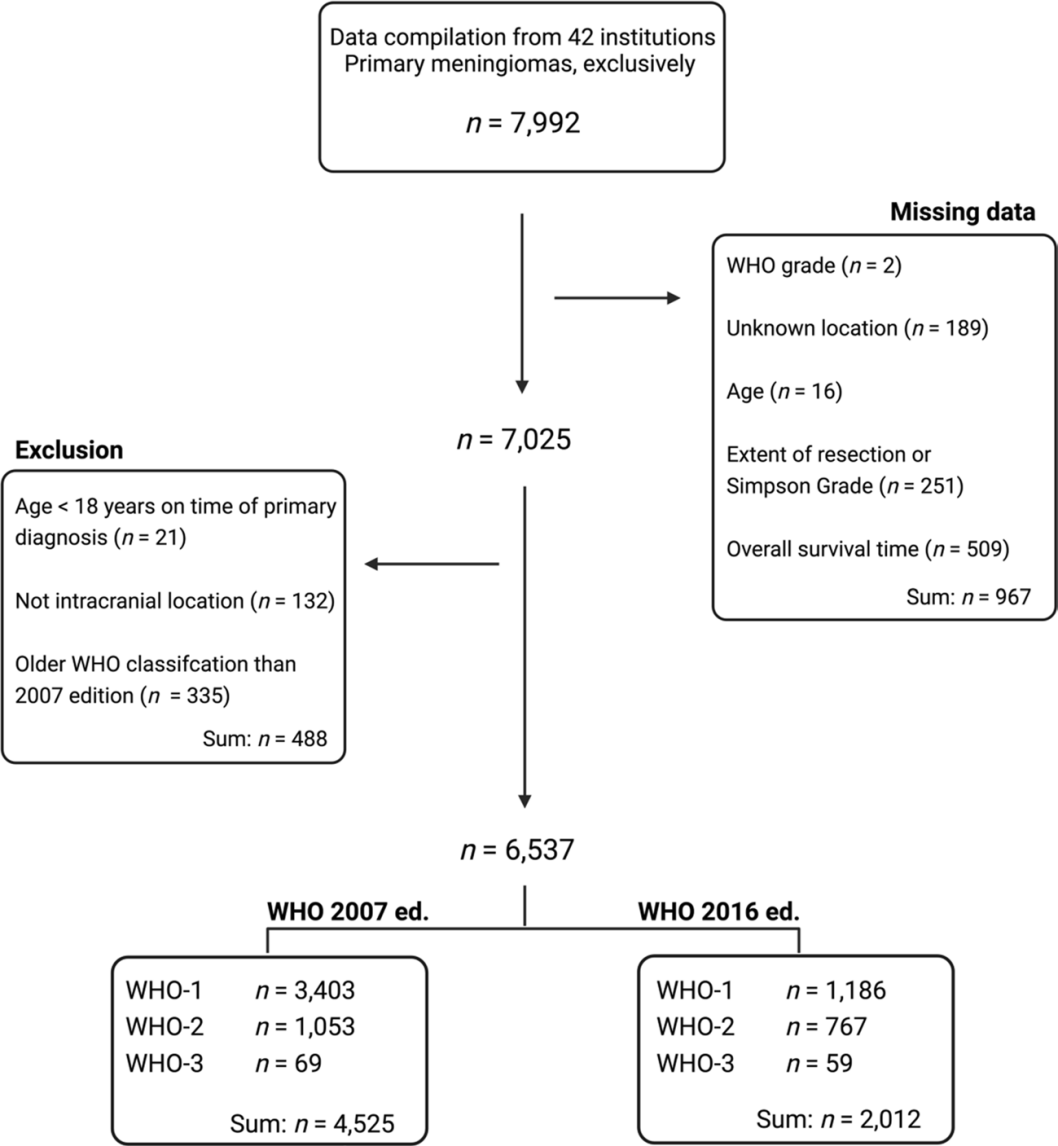
Flowchart of patients included from the PERNS database

Using our PERNS database, eligible cases comprised meningioma patients without missing information on event history, WHO grading, age, location or extent of resection. The following inclusion criteria were imposed: at least 18-years of age at time of entry, intracranial tumor location, and either the 2007 or 2016 edition of the WHO classification was applied in diagnostics. Patients were excluded if they did not have relevant data present (Fig. 1).

### Statistics analysis of the *PERNS* data

The included cases were followed from day of surgical removal of the primary lesion. Follow-up continued until recurrence, death, last recorded data in the local registry, or a maximum of 10 years post-surgery, whichever occurred first.

For the Kaplan–Meier analysis of time to recurrence, patients who died without a recurrence were censored at the time of their death. Hence, the data of each patient consisted of a time to one of two possible observations *and* an indicator of which of the two observations occurred—the usual survival data format for Kaplan–Meier analysis [7]. The two possible observations were either (1) recurrence has occurred at the recorded time or (2) follow-up ended at the recorded time and a recurrence has not occurred. In the latter case the censored time was therefore either a time to loss of follow-up or a time to death. No distinction is made between loss of follow-up and death. Unlike the Kaplan–Meier method, the Aalen-Johansen method does not disregard information that permits differentiation between loss of follow-up and death. Contrarily, the Aalen-Johansen method recognizes that patients who die can no longer experience a recurrence. Accordingly, the data required for each patient in the Aalen-Johansen analysis consist of a time to one of three possible observations *and* an indicator of which observations occurred—the usual data format for competing risk analysis [7]. The three possible observations should be (1) a recurrence has occurred at the recorded time, (2) a recurrence-free death has occurred at the recorded time and (3) follow-up ended at the recorded time and neither a recurrence nor a death has occurred. In the latter case, the censored time corresponds to a time to loss of follow-up at which the patient is known to be recurrence-free and alive (i.e., still “at risk”).

Herein, we used the free statistical software R v. 4.3.0 and the package “prodlim” v. 2023–03-31 for the competing risk analysis.

## Results

From the PubMed survey, we identified 513 papers that were published since 2020. Of these, 179 were eligible for full-text screening. Of all available studies, 10 studies did not report the applied methodology for risk estimation, thereby reducing to 169 relevant papers. One hundred and fifty-nine studies (88.8% of 169) used the Kaplan–Meier method and 10 (5.9% of 169) considered competing risk analysis using the Aalen-Johansen method (Table 1 and Supplementary Table 1 for detailed overview).

**Table 1:** Summary on methodology applied to estimate risk of recurrence in studies published between 2020 and primo-2023 (*n* = 169). Interquartile range (IQR) denoted the range between 1st and 3rd quartile. The references of these studies are included in Supplementary Table 1

Method used for risk estimation	% of WHO grades in each study Median of all percentages summarized	Median of patients included (IQR)
Kaplan–Meier 159 (94.1%)	WHO1: 61.6% (0 to 89.6) WHO2: 15.0% (0 to 55.8) WHO3: 0% (0 to 8.6)	*n* = 122 (46.0 to 258.0)
Aalen-Johansen 10 (5.9%)	WHO1: 57.8% (27.9 to 91.0) WHO2: 18.0% (6.0 to 31.0) WHO3: 2.4% (1.6 to 26.1)	*n* = 162 (80 to 618)

### Eligible cases

A total of 6,537 meningioma patients from our PERNS database were included in the study (Fig. 1). The cohort was followed for 32,755 person-years with a median follow-up of 50.5 months (the interquartile ranged from 24.0 to 89.0 months). There were 5,093 patients censored alive and recurrence-free, a total of 1,113 with recurrence, and 331 recurrence-free deaths within 10 years postoperatively. There was no loss of patients during follow-up.

### Risk of recurrence

**Fig. 2:**
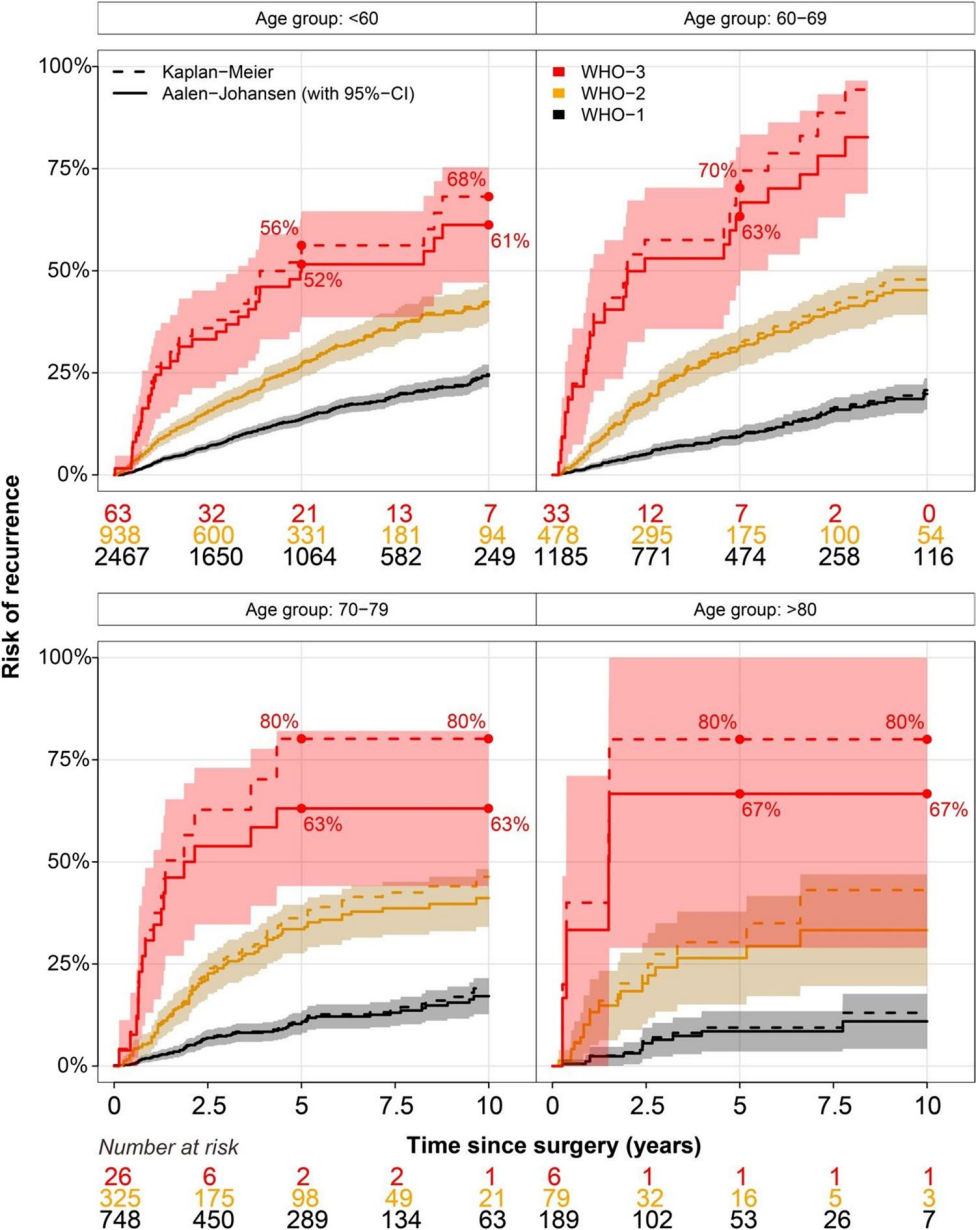
The risk of recurrence when estimated with the Kaplan–Meier *vs.* Aalen-Johansen method while stratified for age group and WHO grade

Figure 2 delineates the risk of recurrence obtained by the Kaplan–Meier and Aalen-Johansen methods, stratified by age groups and WHO grade. The discrepancy between the two methods increased gradually with higher age and higher WHO grade, as the number of competing events increased correspondingly in these subgroups. Specifically, the 10-year risk of recurrence was 24.6% (95% CI: 21.8 to 27.5) using Kaplan–Meier (1 – Kaplan–Meier) *vs*. 24.2% (95% CI: 21.5 to 27.0) using the Aalen-Johansen method. In the group of patients aged 70 to 79 years who have a WHO-3 lesion, however, the Kaplan–Meier method estimated 80.1% (95% CI: 58.8 to 100.0) risk of recurrence at 10 years after surgery. In contrast, the estimate obtained from Aalen-Johansen method was 63.1% (95% CI: 44.1 to 82.1) risk of recurrence at the same timepoint. Similar observations were made for the group of patients older than 80 and a WHO-3 lesion. Supplementary Table 2 provides the results obtained with the two methods at time points 2.5, 5.0, 7.5, and 10 years.

## Discussion

The present study and literature review indicate low awareness regarding the importance of considering competing risks in the analysis of meningioma recurrence. Approximately 94% of recent articles on meningioma recurrences used a Kaplan–Meier method and did not consider competing risks analyses. From a methodological point of view, the selected approach was incorrect, and it can be concluded that the risk of recurrence was overestimated in these studies. In cases of competing events, the need of using the Aalen-Johansen method is to avoid bias inherent from the Kaplan–Meier method, which has been well-documented in both statistical and medical journals [1–10].

Our illustrative example highlighted a relatively minor discrepancy in the risk estimates derived from the two methods when applied to patients with benign phenotypes and younger ages. The discrepancy became more pronounced in subgroups comprising older patients with aggressive phenotypes, particularly those with WHO-3 meningiomas. These findings illustrate well-documented pitfalls associated with the inappropriate application of the Kaplan–Meier estimator [2]. Specifically, the erroneous overestimation will increase with the number of competing events observed within each subgroup. In our study, this was particularly relevant to recurrence-free deaths, which were observed more frequently in patients with advanced age and those with high-grade lesions [2]. In contrast, when few competing events are present—as seen in cases of low-grade lesions in younger patients with very few recurrence-free deaths—the estimates from both methods tend to converge. In scenarios where there are no competing events, the Aalen-Johansen analysis effectively becomes identical to a Kaplan–Meier analysis, yielding the same results [2]. The similarity of results between the incorrect Kaplan–Meier and the correct Aalen-Johansen method in some subgroups might lead one to question the significance of choosing the correct statistical approach. Although incorrect statistical methods may sometimes produce correct results by chance, it is always erroneous to claim that these results are reliably supported by the data analysis when the chosen methods are inappropriate. It is self-evident that reliable scientific methodology cannot include questionable statistical analyses.

Based on our findings, we pragmatically concluded that there is no need to recalculate results regarding patients with a WHO-1 or -2 meningioma, especially in younger patients, from older studies that used the Kaplan-Meir estimator. The analysis of our *PERNS* data showed that the competing risk of death was low in these subgroups and the incorrect Kaplan–Meier method overestimated recurrences only to a neglible extent. We believe this finding can be generalized. The bias from incorrect methodology is probably neglible for all previously published results obtained by the Kaplan–Meier method and used on data from patients with a WHO-1 and -2 meningiomas. In contrast, our analysis of older patients with aggressive phenotypes, primarily WHO-3 meningiomas, indicated substantially lower risks of recurrence when the competing event of dying before a recurrence was properly accounted for, i.e. using the Aalen-Johansen method. This is because the competing risk of death was observed more frequently in this subgroup. These results are important in guiding therapeutic decisions for aggressive lesions in older patients. However, it was not within our scope to address this further, and it must be emphasized that these categories with high-grade lesions in older individuals comprised relatively small subgroups, thus necessitating further research. Nevertheless, this specific finding underscores the relevance of competing risk analysis. A systematic overestimation of the recurrence risk could affect evidence-based decision-making by misguiding clinicians and potentially exposing patients to unnecessary immediate morbidity from surgery, radiotherapy or experimental medication. Individual management with surgery and adjuvant treatment aims to balance the risk of immediate morbidity due to more aggressive treatment options with the risk of morbidity from recurrences after conservative management. Aggressive treatment is sometimes necessary, but overly aggressive surgery and radiation with subsequent risks of morbidity may not be in the best interest for patients if the risk of recurrence is substantially lower than suggested in the literature. Further, an overly aggressive therapy can increase the competing risk of death itself. Therefore, aggressive therapy leading to an increase in the competing event of death may lower the risk of recurrence; however, this is not attributed to an anti-tumorigenic effect but rather because death prevents the recurrence. Hence, it is warranted and important to also report the competing risk of recurrence-free death in addition to the risk of recurrence [11]. Not only recurrences but also survival must be considered when informing patients and making treatment decisions.

We demonstrate that the Kaplan–Meier method was dominating in meningioma research published during the last years, which also agrees with observations from older literature [12–14]. However, adoption of competing risk analysis is the only guarantee to ensure correct risk estimates regardless of age and phenotype. We can see no reason to avoid competing risk analysis when estimating risk of recurrence in meningioma patients. The interpretation of risk estimates obtained from the Aalen-Johansen method is the same as when the Kaplan–Meier method is used appropriately (i.e. in scenarios without competing risks), and the statistical software required for the data analysis are widely accessible and straightforward to use.

A strength of this paper was the comprehensive meningioma cohort, with data from 6,537 meningioma patients collected from 42 international centers. The results are, therefore, generalizable to a large extent, as they reflect a global cohort.

There are several limitations to the current study. Our analysis included patients classified according to the 2007 or 2016 edition of the WHO Classification, exclusively, while advancements in molecular biomarker and epigenetic classifications have since been introduced [13]. Misclassification of some patients might have occurred per criteria in the 2021 edition of the WHO Classification. Any misclassification due to incorrect categorization based on molecular or epigenetic characteristics will impact the results, regardless of the statistical method used to analyze the data. However, due to specific molecular features, such as *TERT* gene alterations and CDKN2A/B homozygous deletions, is likely to impact only a minor fraction of our cohort [15, 16]. In the present study, our primary objective was to demonstrate the implications of overlooking competing risks in the recurrence analysis of meningiomas. The reclassification of even a small proportion of cases would not significantly alter our overreachig conclusions. Finally, it was not our aim to present detailed statistical results related to clinical research questions, but to describe whether competing risk analysis were appropriately utilized in meningioma research when indicated and to what methodological inaccuracies could skew the analysis of recurrence risk.

The analyses performed are not exhaustive, which can be considered a weakness. There is a variety of methods to analyze competing risk data; only one, maybe the simplest and most often relevant, was discussed here. Our aim was not to provide a profound tutorial on competing risks analysis but to make a basic point and promote a simple method that could easily lead to improvement in meningioma research. The following references provide excellent tutorials on competing risks data analysis [1–7, 9, 10]. Moreover, we studied meningiomas, although the discordance between Kaplan–Meier and competing risk analyses applies to all analyses with competing outcomes. We hypothesize that similar observations could be made for other brain tumors, although we focused on the need to reanalyze meningioma data.

## Conclusion

The Kaplan–Meier method is customary in meningioma research but systematically overestimates the risk of recurrence because of the competing risk of recurrence-free death. The Aalen-Johansen method offers a simple alternative that better accounts for competing risks. We observed that discrepancy between two methods was most pronounced among the oldest patients with higher grade tumors. Adopting competing risk analysis is a straightforward change in statistical methodology that could reduce the risk of bias in future publications.

### Supplementary Information

Below is the link to the electronic supplementary material.

Supplementary file1 (DOCX 1139 KB)

Supplementary file2 (DOCX 21 KB)
