# Supplementary material for: The importance of considering competing risks in recurrence analysis of intracranial meningioma
Source: J Neurooncol. 2024 Feb 10;166(3):503–11. doi: 10.1007/s11060-024-04572-y (PMC10876814; doi:10.1007/s11060-024-04572-y)
Supplement: Supplementary file 1 — Supplementary file1 (DOCX 1139 KB) [file 11060_2024_4572_MOESM1_ESM.docx]

**The importance of considering competing risks in recurrence analysis of intracranial meningioma**

Christian Mirian^1 (ORC ID: 0000-0001-6801-0123)^, Lasse Rehné Jensen^1 (ORC ID: 0000-0001-6931-4399)^ , Tareq A. Juratli^2,3 (ORC ID: 0000-0003-2236-6719)^, Andrea Daniela Maier^1,4 (ORC ID: 0000-0002-5930-0636)^, Sverre H. Torp^5,6^, Helen A. Shih^7^, Ramin A. Morshed^8^, Jacob S. Young^8 (ORC ID: 0000-0002-5499-4325)^, Stephen T. Magill^8,9 (ORC ID: 0000-0002-5257-7835)^, Luca Bertero^10 (ORC ID: 0000-0001-9887-7668)^, Walter Stummer^11^, Dorothee Cäcilia Spille^11^, Benjamin Brokinkel^11, 12^, Soichi Oya^13^, Satoru Miyawaki^14^, Nobuhito Saito^14^, Martin Proescholdt^15^, Yasuhiro Kuroi^16^, Konstantinos Gousias^17^, Matthias Simon^18^, Jennifer Moliterno^19^, Ricardo Prat-Acin^20^, Stéphane Goutagny^21^, Vikram C. Prabhu^22^, John T. Tsiang^22 (ORC ID: 0000-0001-5869-6408)^, Johannes Wach^23^, Erdem Güresir^23^, Junkoh Yamamoto^24^, Young Zoon Kim^25 (ORC ID: 0000-0003-1171-0780)^, Joo Ho Lee^26^, Matthew Koshy^27^, Karthikeyan Perumal^28^, Mustafa K. Baskaya^28^, Donald M. Cannon^29^, Dennis C. Shrieve^29^, Chang-Ok Suh^30^, Jong Hee Chang^31^, Maria Kamenova^32^, Sven Straumann^32^, Jehuda Soleman^32^, Ilker Y. Eyüpoglu^2^, Tony Catalan^8 (ORC ID: 0009-0005-8821-3880)^, Austin Lui^8 (ORC ID: 0000-0003-1347-8815)^, Philip V. Theodosopoulos^8^, Michael W. McDermott^8,33^, Fang Wang^34^, Fuyou Guo^34^, Pedro Góes^35^, Manoel Antonio de Paiva Neto^35^, Aria Jamshidi^36^, Ricardo Komotar^36^ , Michael Ivan^36^, Evan Luther^36^, Luis Souhami^37^, Marie-Christine Guiot^38^, Tamás Csonka^39^, Toshiki Endo^40^, Olivia Claire Barrett^41^, Randy Jensen^42^, Tejpal Gupta^43^, Akash J. Patel^44,45,46^, Tiemo J. Klisch^46,47^, Jun Won Kim^48^, Francesco Maiuri^49^, Valeria Barresi^50^, María Dolores Tabernero^51 (ORC ID: 0000-0002-4430-9806)^, Simon Skyrman^52^, Anders B. Jørgensen^1^, Mathias Jacobsen Bach^1^, Ian Law^53,54^, David Scheie^4^, Bjarne Winther Kristensen^4,55^, Tina Nørgaard Munch^1,54,56^, Torstein Meling^1,57^, Kåre Fugleholm^1,54^, Paul Blanche^58^, Tiit Mathiesen^1,52,54^

**Corresponding author**

Christian Mirian, MD

[Christian.mirian.larsen@regionh.dk](mailto:Christian.mirian.larsen@regionh.dk)

**Affiliations**

1: Department of Neurosurgery, Copenhagen University Hospital, Copenhagen, Denmark

2: Department of Neurosurgery, Division of Neuro-Oncology, Faculty of Medicine and University Hospital Carl Gustav Carus, Technische Universität Dresden, 01307 Dresden, Germany

3: Department of Neurosurgery, Laboratory of Translational Neuro-Oncology, Massachusetts General Hospital Cancer Center, Harvard Medical School, Boston, USA

4: Department of Pathology, Bartholin Institute, Rigshospitalet, Copenhagen University Hospital, Copenhagen, Denmark

5: Department of Clinical and Molecular Medicine, Faculty of Medicine and Health Sciences, Norwegian, University of Science and Technology (NTNU), Laboratory Centre, St. Olavs hospital, NO-7491 Trondheim, Norway,

6: Department of Pathology, Laboratory Centre, St. Olavs hospital, NO-7030 Trondheim, Norway

7: Department of Radiation Oncology, Massachusetts General Hospital, Harvard Medical School, Boston, MA, USA

8: Department of Neurological Surgery, University of California San Francisco, San Francisco, California, USA

9: Department of Neurological Surgery, Northwestern University, Feinberg School of Medicine, Illinois, USA

10: Pathology Unit, Department of Medical Sciences, University and Città della Salute e della Scienza University Hospital of Turin, Turin, Italy

11: Department of Neurosurgery, University of Münster, Münster, Germany

12: Institute for Neuropathology, University of Münster, Münster, Germany

13: Department of Neurosurgery, Saitama Medical Center/University, Saitama, Japan

14: Department of Neurosurgery, The University of Tokyo Hospital, Tokyo, Japan

15: Department of Neurosurgery, University Regensburg Medical Center, Regensburg, Germany

16: Department of Neurosurgery, Tokyo Women's Medical University, Adachi Medical Center, Tokyo, Japan

17: Department of Neurosurgery, Athens Medical Center, Athens, Greece

18: Department of Neurosurgery, Bethel Clinic University of Bielefeld Medical Center, Bielefeld, Germany

19: Department of Neurosurgery, Yale School of Medicine Yale New Haven Hospital, Smilow Cancer Hospital, New Haven, USA

20: Department of Neurosurgery, Hospital La Fe, Valencia. Spain

21: Université Paris Cité, Department of Neurosurgery, Beaujon Hospital, Assistance Publique Hôpitaux de Paris, Paris, France

22: Department of Neurological Surgery, Loyola University Medical Center, Stritch School of Medicine, Illinois, USA

23: Department of Neurosurgery, University Hospital Leipzig, Leipzig, Germany

24: Department of Neurosurgery, University of Occupational and Environmental Health, Kitakyushu, Japan

25: Department of Neurosurgery, Samsung Changwon Hospital, Sungkyunkwan University School of Medicine, Changwon, Republic of Korea

26: Department of Radiation Oncology, Seoul National University Hospital, Seoul National University College of Medicine, Seoul, Republic of Korea.

27: Department of Radiation Oncology, University of Illinois Hospital and Health Sciences System, Illinois, USA

28: Department of Neurosurgery, University of Wisconsin Medical School & Public Health, Madison, Wisconsin, USA

29: Department of Radiation Oncology Spencer Fox Eccles School of Medicine University of Utah, Utah, USA

30: Department of Radiation Oncology, Yonsei University College of Medicine, Seoul, Republic of Korea

31: Department of Neurosurgery, Yonsei University College of Medicine, Seoul, Republic of Korea

32: Department of Neurosurgery, University Hospital Basel, Basel, Switzerland.

33: Division of Neurosurgery, Miami Neuroscience Institute, Miami, Florida, USA

34: Department of Neurosurgery, The First Affiliated Hospital of Zhengzhou University, Zhengzhou, Henan, China

35: Department of Neurosurgery, Federal University of São Paulo, São Paulo, Brazil

36 Department of Neurological Surgery, Sylvester Comprehensive Cancer Center, University of Miami, Florida, USA

37: Division of Radiation Oncology, McGill University Health Centre, McGill University, Montreal, Quebec, Canada

38: Department of Pathology, McGill University Health Centre, Montreal, Quebec, Canada

39: Department of Pathology, Faculty of Medicine, University of Debrecen, Hungary

40: Division of Neurosurgery, Tohoku Medical and Pharmaceutical University, Tohoku, Japan

41: Infirmary Cancer Care, Alabama, USA

42: Department of Neurosurgery, Huntsman Cancer Institute, University of Utah, Salt Lake City, Utah, USA

43: Department of Radiation Oncology ACTREC, Tata Memorial Centre, HBNI Kharghar, Navi Mumbai:410210, India

44: Department of Neurosurgery, Baylor College of Medicine, Houston, Texas, USA

45: Department of Otolaryngology-Head and Neck Surgery, Baylor College of Medicine, Houston, TX

46: Jan and Dan Duncan Neurological Research Institute, Texas Children’s Hospital, Houston, TX

47: Department of Molecular and Human Genetics, Baylor College of Medicine, Houston, TX, USA.

48: Department of Radiation Oncology, Gangnam Severance Hospital, Yonsei University College of Medicine, Seoul, Republic of Korea

49: Department of Neurosurgery, University of Naples Federico II, Naples, Italy

50: Department of Diagnostics and Public Health, University of Verona, Italy

51: Instituto de Investigación Biomédica de Salamanca (IBSAL), University Hospital of Salamanca, Salamanca, Spain

52: Department of Clinical Neuroscience, Karolinska Institutet, Stockholm, Sweden

53: Department of Clinical Physiology and Nuclear Medicine, Copenhagen University Hospital-Rigshospitalet, Copenhagen, Denmark

54: Department of Clinical Medicine, Faculty of Health and Medical Sciences, University of Copenhagen, Copenhagen, Denmark.

55: Department of Clinical Medicine and Biotech Research and Innovation Center (BRIC), University of Copenhagen, Copenhagen, Denmark

56: Department of Epidemiology Research, Statens Serum Institut, Copenhagen, Denmark

57: Department of Neurological Surgery, Istituto Nazionale Neurologico "C.Besta", Milan, Italy

58: Section of Biostatistics, Department of Public Health, University of Copenhagen, Copenhagen, Denmark.

**Supplementary Table 1. Estimation of risk of recurrence in meningioma literature – review of methodology**

**Day of last review:** 17/02/2023

**Pubmed search string:** Meningioma[MeSH] AND (recurrence[title/abstract] OR progression[title/abstract])

**Years:** In between 2020-2023

**Number of studies:** 513 – 179 deemed relevant

**Excluded:**

- Case-reports,
- No survival analysis methods in study

| **Ref** | **Study, year** | **WHO classification** | **Patients per WHO grade** | **Method** |
| --- | --- | --- | --- | --- |
| [1] | Kopf et al. 2023 | 2016 | WHO-1: 232  WHO-2/3: 35 | Kaplan-Meier |
| [2] | Boetto et al. 2023 | 2016 | WHO-1: 124 | Kaplan-Meier |
| [3] | Brastianos et al. 2022 | 2016 | WHO-1: 12  WHO-2/3: 26 | Kaplan-Meier |
| [4] | Rogers et al. 2023 | 2016 | WHO-1: 60  WHO-2: 51  WHO-3: 53 | Aalen-Johansen |
| [5] | Nguyen et al. 2022 | 2016 | WHO-1: 889 | Kaplan-Meier |
| [6] | Haddad et al. 2022 | 2016 | WHO-1: 129 | Kaplan-Meier |
| [7] | Chotai et al. 2022 | 2016 | WHO-1: 238  WHO-2/3: 115 | Kaplan-Meier |
| [8] | Oya et al. 2022 | 2016 | WHO-1: 4641 | Kaplan-Meier |
| [9] | Thomann et al. 2022 | 2016 | 240 unknown | Kaplan-Meier |
| [10] | Wang et al. 2022 | 2016 | WHO-1: 140  WHO-2: 23  WHO-3: 2 | Kaplan-Meier |
| [11] | Lisowski et al. 2022 | 2016 | WHO-1: 38  WHO-2: 20  WHO-3: 18 | Kaplan-Meier |
| [12] | Zhang et al. 2022 | 2016 | WHO-1: 54  WHO-2: 21  WHO-3: 18 | Kaplan-Meier |
| [13] | Chotai et al. 2022 | 2016 | WHO-1: 137  WHO-2/3: 45 | Kaplan-Meier |
| [14] | Zeng et al. 2022 | 2016 | WHO-2: 98 | Kaplan-Meier |
| [15] | Alexander et al. 2022 | 2016 | WHO-1: 2  WHO-2: 10  WHO-3: 11 | Kaplan-Meier |
| [16] | Pessina et al. 2022 | 2016 | WHO-1: 123 | Kaplan-Meier |
| [17] | Di Bonaventura et al. 2022 | 2016 | WHO-1: 27  WHO-2: 44  WHO-3: 16 | Kaplan-Meier |
| [18] | Jensen et al. 2022 | 2016 | WHO-1: 55  WHO-2: 41  WHO-3: 37 | Aalen-Johansen /Kaplan-Meier |
| [19] | Zhang et al. 2022 | 2016 | 3:38 | Kaplan-Meier |
| [20] | Tang et al. 2022 | 2016 | WHO-1: 31  WHO-2: 6  WHO-3: 10 | Kaplan-Meier |
| [21] | Daza-Ovalle et al. 2022 | 2016 | WHO-1: 9  WHO-2: 1 | Kaplan-Meier |
| [22] | Wujanto et al. 2022 | 2007/2016 | WHO-2: 1078 |  |
| [23] | Inoue et al. 2022 |  | 75 unknown | Kaplan-Meier |
| [24] | Park et al. 2022 | 2016 | WHO-2: 199 | Kaplan-Meier |
| [25] | Fodi et al. 2022 | 2016 | WHO-1: 538  WHO-2: 106  WHO-3: 22 | Kaplan-Meier |
| [26] | Thakur et al. 2022 | 2016 | WHO-1: 173  WHO-2: 19  WHO-3: 1 | Kaplan-Meier |
| [27] | Lin et al. 2022 | 2016 | WHO-2: 94 |  |
| [28] | Dauleac et al. 2022 | 2016 | WHO-1: 48 | Kaplan-Meier |
| [29] | Dedeciusova et al. 2022 | 2016 | WHO-1: 46 | Kaplan-Meier |
| [30] | Pikis et al. 2022 | 2016 | WHO-1: 173 | Kaplan-Meier |
| [31] | Zeng et al. 2022 | 2016 | WHO-2: 260 | Kaplan-Meier |
| [32] | Turner et al | 2016 | WHO-1: 352  WHO-2: 119  WHO-3: 5 | Kaplan-Meier |
| [33] | Teranishi et al | 2016 | WHO-1: 281 | Kaplan-Meier |
| [34] | Preusser et al. 2022 | 2016 | WHO-2: 55  WHO-3: 35 | Kaplan-Meier |
| [35] | Driver et al. 2022 | 2016 | WHO-1: 326  WHO-2: 172  WHO-3: 29 | Kaplan-Meier |
| [36] | Marchetti et al. 2022 | 2016 | WHO-2: 24 | Kaplan-Meier |
| [37] | Chang et al. 2022 | 2016 | WHO-1: 336  WHO-2: 157 | Kaplan-Meier |
| [38] | Rebchuk et al. 2022 | 2016 | WHO-2: 189 | Kaplan-Meier |
| [39] | Anand et al. 2022 | 2016 | WHO-2: 103 | Kaplan-Meier |
| [40] | Daoud et al. 2022 | 2016 | WHO-2: 12 | Kaplan-Meier |
| [41] | Belanger et al. 2022 | 2016 | WHO-1: 2  WHO-2: 8  WHO-3: 2 | Kaplan-Meier |
| [42] | Nidamanuri et al. 2022 | 2016 | WHO-2: 4  WHO-3: 4 | Kaplan-Meier |
| [43] | Champeaux-Depond et al. 2022 | 2016 | WHO-1: 23213  WHO-2: 1548  WHO-3: 596 | Aalen-Johansen |
| [44] | Devalckeneer et al. 2022 | 2016 | WHO-2: 9 | Kaplan-Meier |
| [45] | Lüthge et al. 2022 | 2016 | WHO-1: 1075  WHO-2: 143 | Kaplan-Meier |
| [46] | Mirian et al. 2022 | 2016 | WHO-1: 50  WHO-2: 240  WHO-3: 120 |  |
| [47] | Brastianos et al. 2022 | 2016 | WHO-2: 23  WHO-3: 3 | Kaplan-Meier |
| [48] | Huang et al. 2022 | 2016 | WHO-1: 34  WHO-2: 18  WHO-3: 13 | Kaplan-Meier |
| [49] | Fu et al. 2022 | 2016 | WHO-1: 122 | Kaplan-Meier |
| [50] | Nakasu et al. 2022 | 2016 | WHO-1: 111 | Kaplan-Meier |
| [51] | Rutland et al. 2022 | 2016 | WHO-1: 50  WHO-2: 134 |  |
| [52] | Mantziaris et al. 2022 | 2016 | 417 unknown | Kaplan-Meier |
| [53] | Sheehan et al. 2022 | 2016 | WHO-1: 1117 | Kaplan-Meier |
| [54] | Bi et al. 2022 | 2016 | WHO-2/3: 25 | Kaplan-Meier |
| [55] | Jackson et al. 2022 | 2016 | WHO-1: 264  WHO-2: 38  WHO-3: 2 | Kaplan-Meier |
| [56] | Mohammed et al. 2021 | 2016 | 32 unknown |  |
| [57] | Hasegawa et al. 2021 | 2016 | WHO-2: 68 | Kaplan-Meier |
| [58] | Maas et al. 2021 | 2016 | WHO-1: 235  WHO-2: 217  WHO-3: 62 | Kaplan-Meier |
| [59] | Troude et al. 2021 | 2016 | WHO-2: 8 | Kaplan-Meier |
| [60] | Hong et al. 2021 | 2016 | WHO-1: 37  WHO-2: 8  WHO-3: 1 | Kaplan-Meier |
| [61] | Jia et al. 2021 | 2016 | WHO-2: 6  WHO-3: 8 | Kaplan-Meier |
| [62] | Seaman et al. 2021 | 2016 | WHO-1: 70  WHO-2: 23  WHO-3: 15 |  |
| [63] | Bunevicius et al. 2021 | 2016 | WHO-1: 81  WHO-2/3: 54 | Kaplan-Meier |
| [64] | Bunevicius et al. 2021 | 2016 | WHO-1: 207 | Kaplan-Meier |
| [65] | Liu et al. 2021 | 2016 | WHO-1: 28  WHO-2: 9  WHO-3: 5 | Kaplan-Meier |
| [66] | Schuring-Pereira et al. 2021 | 2016 | WHO-2: 29 | Kaplan-Meier |
| [67] | Biczok et al. 2021 | 2016 | WHO-1: 416 | Kaplan-Meier |
| [68] | Banan et al. 2021 | 2016 | WHO-1: 316  WHO-2: 170 | Kaplan-Meier |
| [69] | Nguyen et al. 2021 | 2016 | WHO-1: 62 | Aalen-Johansen |
| [70] | Unterberger | 2021 | WHO-2: 43 | Kaplan-Meier |
| [71] | Nassiri et al. 2021 | 2016 | WHO-1: 59  WHO-2: 43  WHO-3: 19 | Kaplan-Meier |
| [72] | Güdük et al. 2021 | 2016 | WHO-2: 170 | Kaplan-Meier |
| [73] | Güdük et al. 2021 | 2016 | WHO-1: 1219  WHO-2: 164  WHO-3: 18 | Kaplan-Meier |
| [74] | Zhang et al. 2021 | 2016 | WHO-3: 67 | Kaplan-Meier |
| [75] | Ruzevick et al. 2021 | 2016 | WHO-3: 19 | Kaplan-Meier |
| [76] | Jung et al. 2021 | 2016 | WHO-2: 115  WHO-3: 26 | Kaplan-Meier |
| [77] | Nassiri et al. 2021 | 2016 | WHO-1: 48  WHO-2: 74  WHO-3: 29 | Kaplan-Meier |
| [78] | Behling et al. 2021 | 2016 | WHO-1: 1001  WHO-2: 250  WHO-3: 17 | Kaplan-Meier |
| [79] | Colli et al. 2021 | 2016 | WHO-1: 711  WHO-2: 83  WHO-3: 10 | Kaplan-Meier |
| [80] | Simonetti et al. 2021 | 2016 | WHO-2: 168  WHO-3: 15 | Kaplan-Meier |
| [81] | Dedeciusova et al. 2021 | 2016 | WHO-1: 68 | Kaplan-Meier |
| [82] | Behling et al. 2021 | 2016 | WHO-1: 1251  WHO-2: 295  WHO-3: 25 | Kaplan-Meier |
| [83] | Yamada et al. 2021 | 2016 | WHO-1: 200 | Kaplan-Meier |
| [84] | Graillon et al. 2021 | 2016 | WHO-1: 1  WHO-2: 10  WHO-3: 7 | Kaplan-Meier |
| [85] | Kowalchuk et al. 2021 | 2016 | WHO-2/3: 271 | Kaplan-Meier |
| [86] | Hoffmann et al. 2021 | 2016 | WHO-2: 31 | Kaplan-Meier |
| [87] | Kobayashi et al. 2021 | 2016 | WHO-1: 69  WHO-2: 40  WHO-3: 3 | Kaplan-Meier |
| [88] | Oya et al. 2021 | 2016 | WHO-1: 406 | Kaplan-Meier |
| [89] | Fatima et al. 2021 | 2016 | WHO-1: 47  WHO-2: 4 |  |
| [90] | Krauss et al. 2021 | 2016 | WHO-1: 172  WHO-2: 15  WHO-3: 1 | Kaplan-Meier |
| [91] | Verbruggen et al. 2021 | 2016 | WHO-1: 43  WHO-2: 15 | Aalen-Johansen |
| [92] | Gillespie et al. 2021 | 2016 | WHO-1: 30  WHO-2: 24 | Kaplan-Meier |
| [93] | Bender et al. 2021 | 2016 | WHO-2: 216 | Kaplan-Meier |
| [94] | Delgado-lopez et al. 2021 | 2016 | WHO-1: 12  WHO-2: 1 | Kaplan-Meier |
| [95] | El-Shehaby et al. 2021 | 2016 | WHO-1: 201 | Kaplan-Meier |
| [96] | Lv et al. 2021 | 2016 | WHO-1: 204 | Kaplan-Meier |
| [97] | Brokinkel et al. 2021 | 2016 | WHO-1: 825  WHO-2: 114 | Kaplan-Meier |
| [98] | Youngblood et al. 2021 | 2016 | WHO-1: 390  WHO-2/3: 79 | Kaplan-Meier |
| [99] | Song et al. 2021 | 2016 | WHO-1: 18  WHO-2: 48  WHO-3: 7 | Kaplan-Meier |
| [100] | L van et al. 2021 | 2016 | WHO-1: 17  WHO-2: 48  WHO-3: 21 | Kaplan-Meier |
| [101] | Sa-Marta et al. 2021 | 2016 | WHO-3: 32 | Kaplan-Meier |
| [102] | Liu et al. 2021 | 2016 | WHO-1: 58  WHO-2/3: 16 | Kaplan-Meier |
| [103] | Bashir et al. 2021 | 2016 | WHO-1: 28  WHO-2: 8  WHO-3: 1 |  |
| [104] | Shepard et al. 2021 | 2016 | WHO-2: 233  WHO-3: 38 | Kaplan-Meier |
| [105] | Prat-Acin et al. 2021 | 2016 | WHO-1: 322  WHO-2: 43 | Kaplan-Meier |
| [106] | Momin et al. 2021 | 2016 | WHO-1: 13  WHO-2: 1  WHO-3: 1 | Kaplan-Meier |
| [107] | Bernatz et al. 2021 | 2016 | WHO-2: 56  WHO-3: 7 | Kaplan-Meier |
| [108] | Mirian et al. 2021 | 2016 | WHO-1: 37  WHO-2: 29  WHO-3: 19 | Kaplan-Meier |
| [109] | Spille et al. 2021 | 2016 | WHO-1: 516  WHO-2/3: 49 | Kaplan-Meier |
| [110] | Okano et al. 2021 | 2016 | WHO-1: 243  WHO-2: 26  WHO-3: 0 | Kaplan-Meier |
| [111] | Bunevicius et al. 2021 | 2016 | WHO-1: 124  WHO-2: 2 | Kaplan-Meier |
| [112] | Masalha et al. 2021 | 2016 | WHO-1: 28  WHO-2: 12  WHO-3: 3 | Kaplan-Meier |
| [113] | Nowak-Choi et al. 2021 | 2016 | WHO-1: 299 | Kaplan-Meier |
| [114] | Ruiz-Garcia et al. 2021 | 2016 | WHO-1: 20  WHO-2: 3 | Kaplan-Meier |
| [115] | Champeaux-Depond et al. 2021 | 2016 | WHO-1: 7911  WHO-2: 508  WHO-3: 206 | Aalen-Johansen /cumlative incidence |
| [116] | Ruge et al. 2021 | 2016 | 188 unknown | Kaplan-Meier |
| [117] | Karaaslan et al. 2021 | 2016 | WHO-1: 295 | Kaplan-Meier |
| [118] | Champeaux-Depond et al. 2021 | 2016 | WHO-1: 235  WHO-2: 8  WHO-3: 8 | Kaplan-Meier |
| [119] | Soni et al. 2021 | 2016 | WHO-2: 214 | Kaplan-Meier |
| [120] | Pikis et al. 2021 | 2016 | 37 unknown |  |
| [121] | Soni et al. 2021 | 2016 | WHO-2: 214 | Kaplan-Meier |
| [122] | Maier et al. 2021 | 2016 | WHO-3: 40 | Kaplan-Meier |
| [123] | Lee et al. 2021 | 2016 | WHO-2: 230 | Kaplan-Meier |
| [124] | Chen et al. 2021 | 2016 | WHO-1: 47  WHO-2: 80  WHO-3: 12 | Kaplan-Meier |
| [125] | Garcia-Segura et al. 2020 | 2016 | WHO-2: 181 | Kaplan-Meier |
| [126] | Bashir et al. 2020 | 2016 | WHO-1: 5  WHO-2: 1 | Kaplan-Meier |
| [127] | Helis et al. 2020 | 2016 | WHO-2: 138  WHO-3: 35 | Kaplan-Meier |
| [128] | Dobran et al. 2020 | 2016 | WHO-2: 73 | Kaplan-Meier |
| [129] | Corniola et al. 2020 | 2016 | WHO-1: 440 | Kaplan-Meier |
| [130] | Mirian et al. 2020 | 2016 | WHO-1: 142  WHO-2: 16  WHO-3: 1 | Aalen-Johansen |
| [131] | Kwee et al. 2020 | 2016 | WHO-1: 159  WHO-2: 7 | Kaplan-Meier |
| [132] | Toland et al. 2020 | 2016 | WHO-1: 24  WHO-2: 20  WHO-3: 6 | Kaplan-Meier |
| [133] | Zador et al. 2020 | 2016 | WHO-1: 140  WHO-2: 88  WHO-3: 24 | Kaplan-Meier |
| [134] | Dresser et al. 2020 | 2016 | 120 unknown | Kaplan-Meier |
| [135] | Mooney et al. 2020 | 2016 | WHO-1: 37  WHO-2: 8  WHO-3: 10 | Kaplan-Meier |
| [136] | Samanci et al. 2020 | 2016 | 6 unknown | Kaplan-Meier |
| [137] | Thakur et al. 2020 | 2016 | WHO-1: 106  WHO-2: 19  WHO-3: 1 | Kaplan-Meier |
| [138] | Turner et al. 2020 | 2016 | WHO-1: 368  WHO-2: 119  WHO-3: 5 |  |
| [139] | Ukai et al. 2020 | 2016 | WHO-2: 28 | Kaplan-Meier |
| [140] | Maiuri et al. 2020 | 2016 | WHO-1: 56 | Kaplan-Meier |
| [65] | He et al. 2020 | 2016 | WHO-1: 26  WHO-2: 10  WHO-3: 3 | Kaplan-Meier |
| [141] | Sievers et al. 2020 | 2016 | WHO-1: 502  WHO-2: 7  WHO-3: 19 | Kaplan-Meier |
| [142] | Lippitz et al. 2020 | 2016 | WHO-1: 86 | Kaplan-Meier |
| [143] | Zhang et al. 2020 | 2016 | WHO-3: 52 | Kaplan-Meier |
| [144] | Soni et al. 2020 | 2016 | WHO-1: 35 | Kaplan-Meier |
| [145] | Karimi et al. 2020 | 2016 | WHO-1: 41  WHO-2: 43  WHO-3: 9 | Kaplan-Meier |
| [146] | De oliveira et al. 2020 | 2016 | WHO-1: 67  WHO-2: 17  WHO-3: 1 | Kaplan-Meier |
| [147] | Oh et al. 2020 | 2016 | WHO-2: 28 | Kaplan-Meier |
| [148] | Assi et al. 2020 | 2016 | WHO-1: 135  WHO-2: 39  WHO-3: 21 | Kaplan-Meier |
| [149] | Maiuri et al. 2020 | 2016 | WHO-1: 49  WHO-2: 34 | Kaplan-Meier |
| [150] | Spille et al. 2020 | 2016 | WHO-1: 825  WHO-2/3: 114 | Kaplan-Meier |
| [151] | Corniola et al. 2020 | 2016 | WHO-1: 1352 | Kaplan-Meier |
| [152] | Pou et al. 2020 | 2016 | WHO-1: 60 | Kaplan-Meier |
| [153] | Kim et al. 2020 | 2016 | WHO-2: 26  WHO-3: 6 | Kaplan-Meier |
| [154] | Fioravanzo et al. 2020 | 2016 | WHO-2: 200 | Kaplan-Meier |
| [155] | Hu et al. 2020 | 2016 | WHO-1: 42 | Kaplan-Meier |
| [156] | Hua et al. 2020 | 2016 | WHO-1: 131  WHO-2: 40  WHO-3: 21 | Kaplan-Meier |
| [157] | Mirian et al. 2020 | 2016 | WHO-1: 169  WHO-2: 365  WHO-3: 143 | Aalen-Johansen |
| [158] | Ma et al. 2020 | 2016 | WHO-1: 17  WHO-2: 14  WHO-3: 3 |  |
| [159] | Lemee et al. 2020 | 2016 | WHO-1: 1355 | Kaplan-Meier |
| [160] | Maier et al. 2020 | 2016 | WHO-3: 24 | Kaplan-Meier |
| [161] | Jiang et al. 2002 | 2007 | WHO-2: 22  WHO-3: 4 | Kaplan-Meier |
| [162] | Nakasu et al. 2020 |  | 989 unknown | Kaplan-Meier |
| [163] | Rogers et al. 2020 | 2016 | WHO-2: 19  WHO-3: 22 | Aalen-Johansen |
| [164] | Zhao et al. 2020 | 2016 | WHO-1: 168 | Kaplan-Meier |
| [165] | Dalle et al. 2020 | 2016 | WHO-1: 46  WHO-2: 8 | Kaplan-Meier |
| [166] | Nakazaki et al. 2020 | 2016 | WHO-1: 37 | Kaplan-Meier |
| [167] | Deng et al. 2020 | 2016 | WHO-1: 218  2/3 16 | Kaplan-Meier |
| [168] | Islim et al. 2020 | 2016 | 441 unknown | Kaplan-Meier and Aalen-Johansen |
| [169] | Liu et al. 2020 |  | 5012 unknown |  |
| [170] | Graillon et al. 2020 | 2016 | WHO-1: 2  WHO-2: 10  WHO-3: 8 | Kaplan-Meier |
| [171] | Zhang et al. 2020 | 2016 | WHO-2: 84 | Kaplan-Meier |
| [172] | Kim et al. 2020 | 2016 | WHO-1: 36  WHO-2: 26  WHO-3: 10 | Kaplan-Meier |
| [173] | Gozal et al. 2020 | 2016 | WHO-1: 45  WHO-2: 4 | Kaplan-Meier |
| [174] | Talacchi et al. 2020 | 2016 | 60 unknown | Kaplan-Meier |
| [175] | Samal et al. 2020 | 2016 | WHO-1: 102  WHO-2: 47 | Kaplan-Meier |
| [176] | Karabagli et al. 2020 | 2016 | WHO-2: 95 | Kaplan-Meier |
| [177] | Keric et al. 2020 | 2016 | WHO-2: 258 | Kaplan-Meier |
| [178] | Mooney et al. 2020 | 2016 | WHO-2: 10  WHO-3: 3 | Kaplan-Meier |
| [179] | Lamba et al. 2020 | 2016 | WHO-1: 275  WHO-2: 59 | Kaplan-Meier |

**REFERENCES**

[1] L. Kopf *et al.*, “Prognosis and histology of sporadic synchronous and metachronous meningiomas and comparative analyses with singular lesions,” *Neurosurg. Rev.*, vol. 46, no. 1, p. 55, Feb. 2023, doi: 10.1007/S10143-023-01958-W.

[2] J. Boetto *et al.*, “GAB1 as a Marker of Recurrence in Anterior Skull Base Meningioma,” *Neurosurgery*, vol. 92, no. 2, pp. 391–397, Feb. 2023, doi: 10.1227/NEU.0000000000002209.

[3] P. K. Brastianos *et al.*, “Alliance A071401: Phase II Trial of Focal Adhesion Kinase Inhibition in Meningiomas With Somatic NF2 Mutations,” *J. Clin. Oncol.*, vol. 41, no. 3, pp. 618–628, Jan. 2023, doi: 10.1200/JCO.21.02371.

[4] C. L. Rogers *et al.*, “Low-risk meningioma: Initial outcomes from NRG Oncology/RTOG 0539,” *Neuro. Oncol.*, vol. 25, no. 1, pp. 137–145, Jan. 2023, doi: 10.1093/NEUONC/NOAC137.

[5] M. P. Nguyen *et al.*, “Supervised machine learning algorithms demonstrate proliferation index correlates with long-term recurrence after complete resection of WHO grade I meningioma,” *J. Neurosurg.*, vol. 138, no. 1, pp. 86–94, Jan. 2022, doi: 10.3171/2022.4.JNS212516.

[6] A. F. Haddad *et al.*, “Posterior-based resection of spinal meningiomas: an institutional experience of 141 patients with an average of 28 months of follow-up,” *J. Neurosurg. Spine*, vol. 38, no. 1, pp. 139–146, Jan. 2022, doi: 10.3171/2022.7.SPINE211603.

[7] S. Chotai *et al.*, “Comparison of supratentorial meningioma resection outcomes by dural reconstruction technique,” *J. Neurosurg.*, vol. 138, no. 1, pp. 70–77, Jan. 2022, doi: 10.3171/2022.4.JNS22290.

[8] S. Oya *et al.*, “Male sex and presence of preoperative symptoms are associated with early recurrence of WHO grade I meningiomas after surgical resection: analysis from the nationwide Brain Tumor Registry of Japan,” *Neurosurg. Rev.*, vol. 46, no. 1, Dec. 2022, doi: 10.1007/S10143-022-01907-Z.

[9] P. Thomann *et al.*, “Natural history of meningiomas: a serial volumetric analysis of 240 tumors,” *J. Neurosurg.*, vol. 137, no. 6, pp. 1639–1649, Dec. 2022, doi: 10.3171/2022.3.JNS212626.

[10] B. Wang, G. J. Zhang, Z. Wu, J. T. Zhang, and P. N. Liu, “Surgical outcomes and prognostic factors of parasagittal meningioma: a single-center experience 165 consecutive cases,” *Br. J. Neurosurg.*, vol. 36, no. 6, pp. 756–761, 2022, doi: 10.1080/02688697.2020.1867825.

[11] D. Lisowski *et al.*, “Health-related quality of life and clinical outcome after radiotherapy of patients with intracranial meningioma,” *Sci. Rep.*, vol. 12, no. 1, Dec. 2022, doi: 10.1038/S41598-022-24192-8.

[12] Y. Zhang, X. Wang, M. Shi, Y. Song, J. Yu, and S. Han, “Programmed death ligand 1 and tumor-infiltrating CD8+ T lymphocytes are associated with the clinical features in meningioma,” *BMC Cancer*, vol. 22, no. 1, Dec. 2022, doi: 10.1186/S12885-022-10249-4.

[13] S. Chotai *et al.*, “Matched case-control analysis of outcomes following surgical resection of incidental meningioma,” *J. Neurooncol.*, vol. 160, no. 2, pp. 481–489, Nov. 2022, doi: 10.1007/S11060-022-04167-5.

[14] L. Zeng *et al.*, “Integration of molecular pathology with histopathology to accurately evaluate the biological behaviour of WHO grade 2 meningiomas and patient prognosis,” *J. Neurooncol.*, vol. 160, no. 2, pp. 497–504, Nov. 2022, doi: 10.1007/S11060-022-04170-W.

[15] A. Y. Alexander *et al.*, “The role of bevacizumab for treatment-refractory intracranial meningiomas: a single institution’s experience and a systematic review of the literature,” *Acta Neurochir. (Wien).*, vol. 164, no. 11, pp. 3011–3023, Nov. 2022, doi: 10.1007/S00701-022-05348-X.

[16] F. Pessina *et al.*, “Skull Base Meningiomas: Is Surgical Resection Enough? Outcome Evaluation and Prognostic Factors Analysis in a Single-Center Cohort,” *J. Neurol. Surg. A. Cent. Eur. Neurosurg.*, vol. 83, no. 6, pp. 516–522, Nov. 2022, doi: 10.1055/S-0041-1739204.

[17] R. Di Bonaventura *et al.*, “Dissecting Stemness in Aggressive Intracranial Meningiomas: Prognostic Role of SOX2 Expression,” *Int. J. Mol. Sci.*, vol. 23, no. 19, Oct. 2022, doi: 10.3390/IJMS231911690.

[18] L. R. Jensen *et al.*, “Somatostatin analogues in treatment-refractory meningioma: a systematic review with meta-analysis of individual patient data,” *Neurosurg. Rev.*, vol. 45, no. 5, pp. 3067–3081, Oct. 2022, doi: 10.1007/S10143-022-01849-6.

[19] G. Zhang *et al.*, “Survival Benefit of Prognostic Factors and Treatment in Adult Patients with Recurrent Anaplastic Meningioma: A Retrospective Case Series and Systematic Literature Review,” *World Neurosurg.*, vol. 166, pp. e758–e769, Oct. 2022, doi: 10.1016/J.WNEU.2022.07.099.

[20] A. R. Tang *et al.*, “Outcomes following surgical resection of cystic intracranial meningiomas,” *J. Neurooncol.*, vol. 160, no. 1, pp. 33–40, Oct. 2022, doi: 10.1007/S11060-022-04096-3.

[21] A. Daza-Ovalle, O. Bin-Alamer, J. Flickinger, A. Niranjan, and L. D. Lunsford, “Outcomes after gamma knife radiosurgery for intraventricular meningiomas,” *J. Neurooncol.*, vol. 160, no. 1, pp. 23–31, Oct. 2022, doi: 10.1007/S11060-022-04091-8.

[22] C. Wujanto, T. Y. Chan, Y. Y. Soon, and B. Vellayappan, “Should adjuvant radiotherapy be used in atypical meningioma (WHO grade 2) following gross total resection? A systematic review and Meta-analysis,” *Acta Oncol.*, vol. 61, no. 9, pp. 1075–1083, 2022, doi: 10.1080/0284186X.2022.2116994.

[23] T. Inoue *et al.*, “Effect of high-dose delivery on the attachment of meningiomas in Gamma Knife surgery: a retrospective study,” *Acta Neurochir. (Wien).*, vol. 164, no. 9, pp. 2465–2471, Sep. 2022, doi: 10.1007/S00701-022-05291-X.

[24] C. J. Park *et al.*, “An interpretable radiomics model to select patients for radiotherapy after surgery for WHO grade 2 meningiomas,” *Radiat. Oncol.*, vol. 17, no. 1, Dec. 2022, doi: 10.1186/S13014-022-02090-7.

[25] C. Fodi *et al.*, “The immunohistochemical expression of SSTR2A is an independent prognostic factor in meningioma,” *Neurosurg. Rev.*, vol. 45, no. 4, pp. 2671–2679, Aug. 2022, doi: 10.1007/S10143-021-01651-W.

[26] J. D. Thakur *et al.*, “Critical appraisal of minimally invasive keyhole surgery for intracranial meningioma in a large case series,” *PLoS One*, vol. 17, no. 7, Jul. 2022, doi: 10.1371/JOURNAL.PONE.0264053.

[27] Y. Lin, P. Dai, Q. Lin, and J. Chen, “A Predictive Nomogram for Atypical Meningioma Based On Preoperative Magnetic Resonance Imaging and Routine Blood Tests,” *World Neurosurg.*, vol. 163, pp. e610–e616, Jul. 2022, doi: 10.1016/J.WNEU.2022.04.034.

[28] C. Dauleac, H. A. Leroy, M. A. Karnoub, L. Obled, P. Mertens, and R. Assaker, “Minimally invasive surgery for intradural spinal meningioma: A new standard? A comparative study between minimally invasive and open approaches,” *Neurochirurgie.*, vol. 68, no. 4, pp. 379–385, Jul. 2022, doi: 10.1016/J.NEUCHI.2022.01.006.

[29] M. Dedeciusova, M. Komarc, M. Faouzi, M. Levivier, and C. Tuleasca, “Tumor control and radiobiological fingerprint after Gamma Knife radiosurgery for posterior fossa meningiomas: A series of 46 consecutive cases,” *J. Clin. Neurosci.*, vol. 100, pp. 196–203, Jun. 2022, doi: 10.1016/J.JOCN.2022.04.031.

[30] S. Pikis *et al.*, “Stereotactic Radiosurgery Compared With Active Surveillance for Asymptomatic, Parafalcine, and Parasagittal Meningiomas: A Matched Cohort Analysis From the IMPASSE Study,” *Neurosurgery*, vol. 90, no. 6, pp. 750–757, Jun. 2022, doi: 10.1227/NEU.0000000000001924.

[31] Q. Zeng *et al.*, “Effectiveness of Postoperative Radiotherapy in Patients with Atypical Meningiomas After Gross Total Resection: Analysis of 260 Cases,” *World Neurosurg.*, vol. 162, pp. e580–e586, Jun. 2022, doi: 10.1016/J.WNEU.2022.03.053.

[32] C. P. Turner *et al.*, “Tumour infiltrating lymphocyte density differs by meningioma type and is associated with prognosis in atypical meningioma,” *Pathology*, vol. 54, no. 4, pp. 417–424, Jun. 2022, doi: 10.1016/J.PATHOL.2021.10.002.

[33] Y. Teranishi *et al.*, “Clinical significance of NF2 alteration in grade I meningiomas revisited; prognostic impact integrated with extent of resection, tumour location, and Ki-67 index,” *Acta Neuropathol. Commun.*, vol. 10, no. 1, Dec. 2022, doi: 10.1186/S40478-022-01377-W.

[34] M. Preusser *et al.*, “Trabectedin for recurrent WHO grade 2 or 3 meningioma: A randomized phase II study of the EORTC Brain Tumor Group (EORTC-1320-BTG),” *Neuro. Oncol.*, vol. 24, no. 5, pp. 755–767, May 2022, doi: 10.1093/NEUONC/NOAB243.

[35] J. Driver *et al.*, “A molecularly integrated grade for meningioma,” *Neuro. Oncol.*, vol. 24, no. 5, pp. 796–808, May 2022, doi: 10.1093/NEUONC/NOAB213.

[36] M. Marchetti *et al.*, “Multisession radiosurgery for grade 2 (WHO), high risk meningiomas. A phase II clinical trial,” *J. Neurooncol.*, vol. 157, no. 3, pp. 397–403, May 2022, doi: 10.1007/S11060-022-03978-W.

[37] W. I. Chang *et al.*, “Risk Stratification to Define the Role of Radiotherapy for Benign and Atypical Meningioma: A Recursive Partitioning Analysis,” *Neurosurgery*, vol. 90, no. 5, pp. 619–626, May 2022, doi: 10.1227/NEU.0000000000001904.

[38] A. D. Rebchuk *et al.*, “Survival and Recurrence Outcomes Following Adjuvant Radiotherapy for Grade 2 Intracranial Meningiomas: 13-Year Experience in a Tertiary-Care Center,” *World Neurosurg.*, vol. 161, pp. e748–e756, May 2022, doi: 10.1016/J.WNEU.2022.02.088.

[39] S. K. Anand *et al.*, “Radiopathologic predictors of recurrence in patients with a gross totally resected atypical meningioma,” *Neurol. Res.*, vol. 44, no. 5, pp. 468–474, 2022, doi: 10.1080/01616412.2021.2022915.

[40] E. V. Daoud *et al.*, “Epigenetic and genomic profiling of chordoid meningioma: implications for clinical management,” *Acta Neuropathol. Commun.*, vol. 10, no. 1, Dec. 2022, doi: 10.1186/S40478-022-01362-3.

[41] K. Belanger, T. H. Ung, D. Damek, K. O. Lillehei, and D. R. Ormond, “Concomitant Temozolomide plus radiotherapy for high-grade and recurrent meningioma: a retrospective chart review,” *BMC Cancer*, vol. 22, no. 1, Dec. 2022, doi: 10.1186/S12885-022-09340-7.

[42] P. Nidamanuri and J. Drappatz, “Immune checkpoint inhibitor therapy for recurrent meningiomas: a retrospective chart review,” *J. Neurooncol.*, vol. 157, no. 2, pp. 271–276, Apr. 2022, doi: 10.1007/S11060-022-03979-9.

[43] C. Champeaux-Depond, J. Weller, P. Constantinou, P. Tuppin, and S. Froelich, “Five-year cause-specific survival after meningioma surgery. A nationwide population-based study,” *Neurochirurgie.*, vol. 68, no. 3, pp. 280–288, Apr. 2022, doi: 10.1016/J.NEUCHI.2021.11.003.

[44] A. Devalckeneer *et al.*, “Progestin-related WHO grade II meningiomas behavior-a single-institution comparative case series,” *Neurosurg. Rev.*, vol. 45, no. 2, pp. 1691–1699, Apr. 2022, doi: 10.1007/S10143-021-01708-W.

[45] S. Lüthge *et al.*, “The applicability of established clinical and histopathological risk factors for tumor recurrence during long-term postoperative care in meningioma patients,” *Neurosurg. Rev.*, vol. 45, no. 2, pp. 1635–1643, Apr. 2022, doi: 10.1007/S10143-021-01697-W.

[46] C. Mirian *et al.*, “Implementation of TERT promoter mutations improve prognostication of the WHO classification in meningioma,” *Neuropathol. Appl. Neurobiol.*, vol. 48, no. 3, Apr. 2022, doi: 10.1111/NAN.12773.

[47] P. K. Brastianos *et al.*, “Phase 2 study of pembrolizumab in patients with recurrent and residual high-grade meningiomas,” *Nat. Commun.*, vol. 13, no. 1, Dec. 2022, doi: 10.1038/S41467-022-29052-7.

[48] R. qiang Huang *et al.*, “Overexpression of phosphatidylethanolamine-binding protein 4 (PEBP4) associates with recurrence of meningiomas,” *Clin. Neurol. Neurosurg.*, vol. 214, Mar. 2022, doi: 10.1016/J.CLINEURO.2022.107148.

[49] J. Fu *et al.*, “Primary versus postoperative gamma knife radiosurgery for intracranial benign meningiomas: a matched cohort retrospective study,” *BMC Cancer*, vol. 22, no. 1, Dec. 2022, doi: 10.1186/S12885-022-09321-W.

[50] Y. Nakasu, K. Mitsuya, S. Nakasu, S. Deguchi, and N. Hayashi, “Outcomes of Preserving the Hypertrophic Arachnoid Membrane in the Brain-meningioma Interface: Long-term Follow-up,” *Neurol. Med. Chir. (Tokyo).*, vol. 62, no. 2, pp. 80–88, 2022, doi: 10.2176/NMC.OA.2021-0209.

[51] J. W. Rutland *et al.*, “Association of mutations in DNA polymerase epsilon with increased CD8+ cell infiltration and prolonged progression-free survival in patients with meningiomas,” *Neurosurg. Focus*, vol. 52, no. 2, Feb. 2022, doi: 10.3171/2021.11.FOCUS21592.

[52] G. Mantziaris *et al.*, “Stereotactic radiosurgery versus active surveillance for asymptomatic, skull-based meningiomas: an international, multicenter matched cohort study,” *J. Neurooncol.*, vol. 156, no. 3, pp. 509–518, Feb. 2022, doi: 10.1007/S11060-021-03923-3.

[53] J. Sheehan *et al.*, “An international multicenter matched cohort analysis of incidental meningioma progression during active surveillance or after stereotactic radiosurgery: the IMPASSE study,” *Neuro. Oncol.*, vol. 24, no. 1, pp. 116–124, Jan. 2022, doi: 10.1093/NEUONC/NOAB132.

[54] W. L. Bi *et al.*, “Activity of PD-1 blockade with nivolumab among patients with recurrent atypical/anaplastic meningioma: phase II trial results,” *Neuro. Oncol.*, vol. 24, no. 1, pp. 101–113, Jan. 2022, doi: 10.1093/NEUONC/NOAB118.

[55] H. N. Jackson *et al.*, “Racial and Socioeconomic Disparities in Patients With Meningioma: A Retrospective Cohort Study,” *Neurosurgery*, vol. 90, no. 1, pp. 114–123, Jan. 2022, doi: 10.1227/NEU.0000000000001751.

[56] N. Mohammed *et al.*, “Neurofibromatosis type 2-associated meningiomas: an international multicenter study of outcomes after Gamma Knife stereotactic radiosurgery,” *J. Neurosurg.*, vol. 136, no. 1, pp. 109–114, Jan. 2021, doi: 10.3171/2020.12.JNS202814.

[57] H. Hasegawa *et al.*, “The role of single-fraction stereotactic radiosurgery for atypical meningiomas (WHO grade II): treatment results based on a 25-year experience,” *J. Neurooncol.*, vol. 155, no. 3, pp. 335–342, Dec. 2021, doi: 10.1007/S11060-021-03882-9.

[58] S. L. N. Maas *et al.*, “Integrated Molecular-Morphologic Meningioma Classification: A Multicenter Retrospective Analysis, Retrospectively and Prospectively Validated,” *J. Clin. Oncol.*, vol. 39, no. 34, pp. 3839–3852, Dec. 2021, doi: 10.1200/JCO.21.00784.

[59] L. Troude *et al.*, “Ipsilateral vs controlateral approach in tuberculum sellae meningiomas surgery: a retrospective comparative study,” *Neurosurg. Rev.*, vol. 44, no. 6, pp. 3581–3591, Dec. 2021, doi: 10.1007/S10143-021-01536-Y.

[60] S. Hong, K. Sato, K. Kagawa, and S. Ichi, “The long-term outcome of CyberKnife-based stereotactic radiotherapy for central skull base meningiomas: a single-center experience,” *Neurosurg. Rev.*, vol. 44, no. 6, pp. 3519–3526, Dec. 2021, doi: 10.1007/S10143-021-01535-Z.

[61] Q. Jia *et al.*, “Surgical management of de novo metastatic meningioma of the spine: An underestimated issue for WHO grade II/III meningiomas,” *Clin. Neurol. Neurosurg.*, vol. 210, Nov. 2021, doi: 10.1016/J.CLINEURO.2021.106995.

[62] S. C. Seaman *et al.*, “Case series of sphenoid wing meningioma - What is a maximal safe resection?,” *Neurochirurgie.*, vol. 67, no. 6, pp. 547–555, Nov. 2021, doi: 10.1016/J.NEUCHI.2021.05.001.

[63] A. Bunevicius *et al.*, “Stereotactic Radiosurgery for Olfactory Groove Meningiomas: An International, Multicenter Study,” *Neurosurgery*, vol. 89, no. 5, pp. 784–791, Nov. 2021, doi: 10.1093/NEUROS/NYAB291.

[64] A. Bunevicius *et al.*, “Stereotactic radiosurgery for clinoid meningiomas: a multi-institutional study,” *Acta Neurochir. (Wien).*, vol. 163, no. 10, pp. 2861–2869, Oct. 2021, doi: 10.1007/S00701-021-04972-3.

[65] W. He *et al.*, “Pediatric meningiomas: 10-year experience with 39 patients,” *J. Neurooncol.*, vol. 149, no. 3, pp. 543–553, Sep. 2020, doi: 10.1007/S11060-020-03649-8.

[66] M. Schuring-Pereira *et al.*, “Atypical meningioma: patterns of postradiotherapy recurrences,” *Br. J. Neurosurg.*, vol. 35, no. 5, pp. 591–596, 2021, doi: 10.1080/02688697.2021.1922606.

[67] A. Biczok *et al.*, “Past medical history of tumors other than meningioma is a negative prognostic factor for tumor recurrence in meningiomas WHO grade I,” *Acta Neurochir. (Wien).*, vol. 163, no. 10, pp. 2853–2859, Oct. 2021, doi: 10.1007/S00701-021-04780-9.

[68] R. Banan *et al.*, “The prognostic significance of clinicopathological features in meningiomas: Microscopic brain invasion can predict patient outcome in otherwise benign meningiomas,” *Neuropathol. Appl. Neurobiol.*, vol. 47, no. 6, pp. 724–735, Oct. 2021, doi: 10.1111/NAN.12700.

[69] E. K. Nguyen, G. R. Pond, J. N. Greenspoon, A. C. Whitton, and C. Hann, “Hypofractionated Stereotactic Radiotherapy for the Treatment of Benign Intracranial Meningiomas: Long-Term Safety and Efficacy,” *Curr. Oncol.*, vol. 28, no. 5, pp. 3683–3691, Oct. 2021, doi: 10.3390/CURRONCOL28050314.

[70] A. Unterberger *et al.*, “Adjuvant radiotherapy for atypical meningiomas is associated with improved progression free survival,” *J. Neurol. Sci.*, vol. 428, Sep. 2021, doi: 10.1016/J.JNS.2021.117590.

[71] F. Nassiri *et al.*, “A clinically applicable integrative molecular classification of meningiomas,” *Nature*, vol. 597, no. 7874, pp. 119–125, Sep. 2021, doi: 10.1038/S41586-021-03850-3.

[72] M. Güdük, M. Ş. Ekşi, B. Bozkurt, M. İ. Usseli, A. Erşen Danyeli, and M. N. Pamir, “Treatment and follow-up results of WHO grade II meningiomas,” *J. Clin. Neurosci.*, vol. 91, pp. 354–364, Sep. 2021, doi: 10.1016/J.JOCN.2021.07.022.

[73] M. Güdük, M. Ş. Ekşi, B. Bozkurt, M. İ. Usseli, A. Erşen Danyeli, and M. N. Pamir, “Neurosurgical follow-up and treatment of a series of 26 WHO grade III meningiomas,” *J. Clin. Neurosci.*, vol. 91, pp. 219–225, Sep. 2021, doi: 10.1016/J.JOCN.2021.06.047.

[74] G. S. Zhang *et al.*, “Primary intracranial papillary meningioma: Analysis of factors of prognosis and systematic review,” *J. Clin. Neurosci.*, vol. 91, pp. 118–124, Sep. 2021, doi: 10.1016/J.JOCN.2021.06.025.

[75] J. Ruzevick, A. Gibson, P. Tatman, S. Emerson, and M. Ferreira, “WHO grade III meningioma: De novo tumors show improved progression free survival as compared to secondary progressive tumors,” *J. Clin. Neurosci.*, vol. 91, pp. 105–109, Sep. 2021, doi: 10.1016/J.JOCN.2021.05.060.

[76] M. Jung *et al.*, “The substantial loss of H3K27me3 can stratify risk in grade 2, but not in grade 3 meningioma,” *Hum. Pathol.*, vol. 115, pp. 96–103, Sep. 2021, doi: 10.1016/J.HUMPATH.2021.06.005.

[77] F. Nassiri *et al.*, “Loss of H3K27me3 in meningiomas,” *Neuro. Oncol.*, vol. 23, no. 8, pp. 1282–1291, Aug. 2021, doi: 10.1093/NEUONC/NOAB036.

[78] F. Behling *et al.*, “H3K27me3 loss indicates an increased risk of recurrence in the Tübingen meningioma cohort,” *Neuro. Oncol.*, vol. 23, no. 8, pp. 1273–1281, Aug. 2021, doi: 10.1093/NEUONC/NOAA303.

[79] B. O. Colli *et al.*, “Epidemiological features of meningiomas: a single Brazilian center’s experience with 993 cases,” *Arq. Neuropsiquiatr.*, vol. 79, no. 8, pp. 706–716, Aug. 2021, doi: 10.1590/0004-282X-ANP-2020-0441.

[80] G. Simonetti *et al.*, “Long term follow up in 183 high grade meningioma: A single institutional experience,” *Clin. Neurol. Neurosurg.*, vol. 207, Aug. 2021, doi: 10.1016/J.CLINEURO.2021.106808.

[81] M. Dedeciusova, M. Majovsky, L. Pecen, V. Benes, and D. Netuka, “Long-term outcome of Simpson IV meningioma resection: Would it improve with adjuvant SRS?,” *Clin. Neurol. Neurosurg.*, vol. 207, Aug. 2021, doi: 10.1016/J.CLINEURO.2021.106766.

[82] F. Behling *et al.*, “The role of Simpson grading in meningiomas after integration of the updated WHO classification and adjuvant radiotherapy,” *Neurosurg. Rev.*, vol. 44, no. 4, pp. 2329–2336, Aug. 2021, doi: 10.1007/S10143-020-01428-7.

[83] S. Yamada *et al.*, “The Impact of 5-Year Tumor Doubling Time to Predict the Subsequent Long-Term Natural History of Asymptomatic Meningiomas,” *World Neurosurg.*, vol. 151, pp. e943–e949, Jul. 2021, doi: 10.1016/J.WNEU.2021.05.023.

[84] T. Graillon *et al.*, “Role of 3D volume growth rate for drug activity evaluation in meningioma clinical trials: the example of the CEVOREM study,” *Neuro. Oncol.*, vol. 23, no. 7, pp. 1139–1147, Jul. 2021, doi: 10.1093/NEUONC/NOAB019.

[85] R. O. Kowalchuk *et al.*, “Treatment of WHO Grade 2 Meningiomas With Stereotactic Radiosurgery: Identification of an Optimal Group for SRS Using RPA,” *Int. J. Radiat. Oncol. Biol. Phys.*, vol. 110, no. 3, pp. 804–814, Jul. 2021, doi: 10.1016/J.IJROBP.2021.01.048.

[86] E. Hoffmann *et al.*, “Retrospective analysis of recurrence patterns and clinical outcome of grade II meningiomas following postoperative radiotherapy,” *Radiat. Oncol.*, vol. 16, no. 1, Dec. 2021, doi: 10.1186/S13014-021-01825-2.

[87] K. Kobayashi *et al.*, “Clinical features and prognostic factors in spinal meningioma surgery from a multicenter study,” *Sci. Rep.*, vol. 11, no. 1, Dec. 2021, doi: 10.1038/S41598-021-91225-Z.

[88] S. Oya *et al.*, “Effect of adjuvant radiotherapy after subtotal resection for WHO grade I meningioma: a propensity score matching analysis of the Brain Tumor Registry of Japan,” *J. Neurooncol.*, vol. 153, no. 2, pp. 351–360, Jun. 2021, doi: 10.1007/S11060-021-03775-X.

[89] N. Fatima, J. H. Shin, W. T. Curry, S. D. Chang, and A. Meola, “Microsurgical resection of foramen magnum meningioma: multi-institutional retrospective case series and proposed surgical risk scoring system,” *J. Neurooncol.*, vol. 153, no. 2, pp. 331–342, Jun. 2021, doi: 10.1007/S11060-021-03773-Z.

[90] W. E. Krauss *et al.*, “Clinical characteristics and management differences for grade II and III spinal meningiomas,” *J. Neurooncol.*, vol. 153, no. 2, pp. 313–320, Jun. 2021, doi: 10.1007/S11060-021-03771-1.

[91] L. C. Verbruggen *et al.*, “Clinical characteristics of subsequent histologically confirmed meningiomas in long-term childhood cancer survivors: A Dutch LATER study,” *Eur. J. Cancer*, vol. 150, pp. 240–249, Jun. 2021, doi: 10.1016/J.EJCA.2021.03.021.

[92] C. S. Gillespie *et al.*, “The growth rate and clinical outcomes of radiation induced meningioma undergoing treatment or active monitoring,” *J. Neurooncol.*, vol. 153, no. 2, pp. 239–249, Jun. 2021, doi: 10.1007/S11060-021-03761-3.

[93] L. Bender *et al.*, “High risk of recurrence for grade II meningioma: a 10-year multicenter analysis of prognosis factors,” *Chinese Clin. Oncol.*, vol. 10, no. 3, pp. 1–9, 2021, doi: 10.21037/CCO-20-226.

[94] P. D. Delgado-López *et al.*, “Volumetric growth rate of incidental asymptomatic meningiomas: a single-center prospective cohort study,” *Acta Neurochir. (Wien).*, vol. 163, no. 6, pp. 1665–1675, Jun. 2021, doi: 10.1007/S00701-021-04815-1.

[95] A. M. N. El-Shehaby, W. A. Reda, K. M. Abdel Karim, A. M. Nabeel, R. M. Emad Eldin, and S. R. Tawadros, “Single-Session Stereotactic Radiosurgery for Large Benign Meningiomas: Medium-to Long-Term Results,” *World Neurosurg.*, vol. 150, pp. e324–e336, Jun. 2021, doi: 10.1016/J.WNEU.2021.03.003.

[96] P. Lv *et al.*, “Long-term outcome in meningiomas involving the major dural sinuses with combined therapy of subtotal resection and early postoperative gamma knife radiosurgery,” *Acta Neurochir. (Wien).*, vol. 163, no. 6, pp. 1677–1685, Jun. 2021, doi: 10.1007/S00701-021-04766-7.

[97] B. Brokinkel *et al.*, “The Simpson grading: defining the optimal threshold for gross total resection in meningioma surgery,” *Neurosurg. Rev.*, vol. 44, no. 3, pp. 1713–1720, Jun. 2021, doi: 10.1007/S10143-020-01369-1.

[98] M. W. Youngblood *et al.*, “Associations of meningioma molecular subgroup and tumor recurrence,” *Neuro. Oncol.*, vol. 23, no. 5, pp. 783–794, May 2021, doi: 10.1093/NEUONC/NOAA226.

[99] J. Song *et al.*, “Radiation-induced brain injury in patients with meningioma treated with proton or photon therapy,” *J. Neurooncol.*, vol. 153, no. 1, pp. 169–180, May 2021, doi: 10.1007/S11060-021-03758-Y.

[100] T. Le Van *et al.*, “Multimodal management of surgery- and radiation-refractory meningiomas: an analysis of the French national tumor board meeting on meningiomas cohort,” *J. Neurooncol.*, vol. 153, no. 1, pp. 55–64, May 2021, doi: 10.1007/S11060-021-03741-7.

[101] E. Sá-Marta, J. L. Alves, O. Rebelo, and M. Barbosa, “World Health Organization Grade III Meningiomas: A Retrospective Study at an Academic Medical Center,” *World Neurosurg.*, vol. 149, pp. e877–e893, May 2021, doi: 10.1016/J.WNEU.2021.01.080.

[102] P. C. Liu, A. S. Lieu, C. J. Lin, H. P. Tsai, C. Y. Chai, and A. L. Kwan, “High Expression of Sp1 is Associated with Recurrence of Meningioma,” *World Neurosurg.*, vol. 149, pp. e1056–e1060, May 2021, doi: 10.1016/J.WNEU.2021.01.016.

[103] A. Bashir, V. A. Larsen, M. Ziebell, K. Fugleholm, and I. Law, “Improved Detection of Postoperative Residual Meningioma with [68Ga]Ga-DOTA-TOC PET Imaging Using a High-resolution Research Tomograph PET Scanner,” *Clin. Cancer Res.*, vol. 27, no. 8, pp. 2216–2225, Apr. 2021, doi: 10.1158/1078-0432.CCR-20-3362.

[104] M. J. Shepard *et al.*, “Stereotactic Radiosurgery for Atypical (World Health Organization II) and Anaplastic (World Health Organization III) Meningiomas: Results From a Multicenter, International Cohort Study,” *Neurosurgery*, vol. 88, no. 5, pp. 980–988, May 2021, doi: 10.1093/NEUROS/NYAA553.

[105] R. Prat-Acín, M. J. Guarín-Corredor, I. Galeano-Senabre, A. Ayuso-Sacido, and F. Vera-Sempere, “Value of KI-67/MIB-1 labeling index and simpson grading system to predict the recurrence of who grade I intracranial meningiomas compared to who grade II,” *J. Clin. Neurosci.*, vol. 86, pp. 32–37, Apr. 2021, doi: 10.1016/J.JOCN.2021.01.009.

[106] A. A. Momin *et al.*, “Outcomes of salvage radiation for recurrent world health organization grade II meningiomas: a retrospective cohort study,” *J. Neurooncol.*, vol. 152, no. 2, pp. 373–382, Apr. 2021, doi: 10.1007/S11060-021-03711-Z.

[107] S. Bernatz *et al.*, “Influence of VEGF-A, VEGFR-1-3, and neuropilin 1-2 on progression-free: and overall survival in WHO grade II and III meningioma patients,” *J. Mol. Histol.*, vol. 52, no. 2, pp. 233–243, Apr. 2021, doi: 10.1007/S10735-020-09940-2.

[108] C. Mirian *et al.*, “Somatostatin Receptor-Targeted Radiopeptide Therapy in Treatment-Refractory Meningioma: Individual Patient Data Meta-analysis,” *J. Nucl. Med.*, vol. 62, no. 4, pp. 507–513, Apr. 2021, doi: 10.2967/JNUMED.120.249607.

[109] D. C. Spille *et al.*, “Predicting the risk of postoperative recurrence and high-grade histology in patients with intracranial meningiomas using routine preoperative MRI,” *Neurosurg. Rev.*, vol. 44, no. 2, pp. 1109–1117, Apr. 2021, doi: 10.1007/S10143-020-01301-7.

[110] A. Okano *et al.*, “Associations of pathological diagnosis and genetic abnormalities in meningiomas with the embryological origins of the meninges,” *Sci. Rep.*, vol. 11, no. 1, Dec. 2021, doi: 10.1038/S41598-021-86298-9.

[111] A. Bunevicius *et al.*, “Stereotactic Radiosurgery for Perioptic Meningiomas: An International, Multicenter Study,” *Neurosurgery*, vol. 88, no. 4, pp. 828–837, Apr. 2021, doi: 10.1093/NEUROS/NYAA544.

[112] W. Masalha *et al.*, “Metabolic alterations in meningioma reflect the clinical course,” *BMC Cancer*, vol. 21, no. 1, Dec. 2021, doi: 10.1186/S12885-021-07887-5.

[113] K. Nowak-Choi *et al.*, “Resected WHO grade I meningioma and predictors of local control,” *J. Neurooncol.*, vol. 152, no. 1, pp. 145–151, Mar. 2021, doi: 10.1007/S11060-020-03688-1.

[114] H. Ruiz-Garcia *et al.*, “Convexity Meningiomas in Patients with Neurofibromatosis Type 2: Long-Term Outcomes After Gamma Knife Radiosurgery,” *World Neurosurg.*, vol. 146, pp. e678–e684, Feb. 2021, doi: 10.1016/J.WNEU.2020.10.153.

[115] C. Champeaux-Depond, P. Constantinou, and J. Weller, “Cause-Specific Survival After Meningioma Surgery: A Nationwide Population-Based Competing Risk Study,” *World Neurosurg.*, vol. 146, pp. e67–e75, Feb. 2021, doi: 10.1016/J.WNEU.2020.10.012.

[116] M. I. Ruge *et al.*, “Stereotactic radiosurgery for treating meningiomas eligible for complete resection,” *Radiat. Oncol.*, vol. 16, no. 1, Dec. 2021, doi: 10.1186/S13014-021-01748-Y.

[117] B. Karaaslan *et al.*, “Stereotactic Radiosurgery after Subtotal Resection of Critically-Located Grade I Meningioma: A Single-Center Experience and Review of Literature,” *Turk. Neurosurg.*, vol. 31, no. 4, pp. 519–529, 2021, doi: 10.5137/1019-5149.JTN.30181-20.2.

[118] C. Champeaux-Depond and J. Weller, “Tamoxifen. A treatment for meningioma?,” *Cancer Treat. Res. Commun.*, vol. 27, Jan. 2021, doi: 10.1016/J.CTARC.2021.100343.

[119] P. Soni *et al.*, “Clear cell histology portends a worse prognosis than other WHO grade II histologies,” *J. Neurooncol.*, vol. 151, no. 2, pp. 307–312, Jan. 2021, doi: 10.1007/S11060-020-03668-5.

[120] S. Pikis, A. Bunevicius, and J. Sheehan, “Outcomes from treatment of asymptomatic skull base meningioma with stereotactic radiosurgery,” *Acta Neurochir. (Wien).*, vol. 163, no. 1, pp. 83–88, Jan. 2021, doi: 10.1007/S00701-020-04648-4.

[121] P. Soni *et al.*, “Extent of resection and survival outcomes in World Health Organization grade II meningiomas,” *J. Neurooncol.*, vol. 151, no. 2, pp. 173–179, Jan. 2021, doi: 10.1007/S11060-020-03632-3.

[122] A. D. Maier *et al.*, “TERT promoter mutations in primary and secondary WHO grade III meningioma,” *Brain Pathol.*, vol. 31, no. 1, pp. 61–69, Jan. 2021, doi: 10.1111/BPA.12892.

[123] G. Lee *et al.*, “Adjuvant Radiation Therapy Versus Surveillance After Surgical Resection of Atypical Meningiomas,” *Int. J. Radiat. Oncol. Biol. Phys.*, vol. 109, no. 1, pp. 252–266, Jan. 2021, doi: 10.1016/J.IJROBP.2020.08.015.

[124] W. C. Chen *et al.*, “A Prognostic Gene-Expression Signature and Risk Score for Meningioma Recurrence After Resection,” *Neurosurgery*, vol. 88, no. 1, pp. 202–210, Jan. 2020, doi: 10.1093/NEUROS/NYAA355.

[125] M. E. Garcia-Segura, A. W. Erickson, R. Jairath, D. G. Munoz, and S. Das, “Necrosis and Brain Invasion Predict Radio-Resistance and Tumor Recurrence in Atypical Meningioma: A Retrospective Cohort Study,” *Neurosurgery*, vol. 88, no. 1, pp. E42–E48, Jan. 2020, doi: 10.1093/NEUROS/NYAA348.

[126] A. Bashir *et al.*, “PET imaging of meningioma with 18F-FLT: a predictor of tumour progression,” *Brain*, vol. 143, no. 11, pp. 3308–3317, 2020, doi: 10.1093/BRAIN/AWAA267.

[127] C. A. Helis *et al.*, “Stereotactic Radiosurgery for Atypical and Anaplastic Meningiomas,” *World Neurosurg.*, vol. 144, pp. e53–e61, Dec. 2020, doi: 10.1016/J.WNEU.2020.07.211.

[128] M. Dobran *et al.*, “Surgical Treatment and Predictive Factors for Atypical Meningiomas: A Multicentric Experience,” *World Neurosurg.*, vol. 144, pp. e1–e8, Dec. 2020, doi: 10.1016/J.WNEU.2020.03.201.

[129] M. V. Corniola, J. M. Lemée, K. Schaller, and T. R. Meling, “Lateral sphenoid wing meningiomas without bone invasion-still skull base surgery?,” *Neurosurg. Rev.*, vol. 43, no. 6, pp. 1547–1553, Dec. 2020, doi: 10.1007/S10143-019-01181-6.

[130] C. Mirian *et al.*, “The Ki-67 Proliferation Index as a Marker of Time to Recurrence in Intracranial Meningioma,” *Neurosurgery*, vol. 87, no. 6, pp. 1289–1298, Dec. 2020, doi: 10.1093/NEUROS/NYAA226.

[131] L. E. Kwee, B. S. Harhangi, G. A. Ponne, J. M. Kros, C. M. F. Dirven, and R. Dammers, “Spinal meningiomas: Treatment outcome and long-term follow-up,” *Clin. Neurol. Neurosurg.*, vol. 198, Nov. 2020, doi: 10.1016/J.CLINEURO.2020.106238.

[132] A. Toland *et al.*, “Pediatric meningioma: a clinicopathologic and molecular study with potential grading implications,” *Brain Pathol.*, vol. 30, no. 6, pp. 1134–1143, Nov. 2020, doi: 10.1111/BPA.12884.

[133] Z. Zador, A. P. Landry, B. Haibe-Kains, and M. D. Cusimano, “Meta-gene markers predict meningioma recurrence with high accuracy,” *Sci. Rep.*, vol. 10, no. 1, Dec. 2020, doi: 10.1038/S41598-020-74482-2.

[134] L. Dresser *et al.*, “Estrogen hormone replacement therapy in incidental intracranial meningioma: a growth-rate analysis,” *Sci. Rep.*, vol. 10, no. 1, Dec. 2020, doi: 10.1038/S41598-020-74344-X.

[135] M. A. Mooney *et al.*, “Is Falcine Meningioma a Diffuse Disease of the Falx? Case Series and Analysis of a ‘Grade Zero’ Resection,” *Neurosurgery*, vol. 87, no. 5, pp. 900–909, Nov. 2020, doi: 10.1093/NEUROS/NYAA038.

[136] Y. Samanci, D. Oktug, M. Yilmaz, M. Sengoz, and S. Peker, “Efficacy of gamma knife radiosurgery in the treatment of intraventricular meningiomas,” *J. Clin. Neurosci.*, vol. 80, pp. 38–42, Oct. 2020, doi: 10.1016/J.JOCN.2020.08.016.

[137] J. D. Thakur *et al.*, “Minimally invasive surgical treatment of intracranial meningiomas in elderly patients (≥ 65 years): outcomes, readmissions, and tumor control,” *Neurosurg. Focus*, vol. 49, no. 4, p. E17, Oct. 2020, doi: 10.3171/2020.7.FOCUS20515.

[138] C. P. Turner, B. van der Werf, A. J. J. Law, A. Bok, M. A. Curtis, and M. Dragunow, “The epidemiology of patients undergoing meningioma resection in Auckland, New Zealand, 2002 to 2011,” *J. Clin. Neurosci.*, vol. 80, pp. 324–330, Oct. 2020, doi: 10.1016/J.JOCN.2020.06.011.

[139] R. Ukai *et al.*, “Recurrence Interval Within 1 Year Leads to Death in Patients with Grade 2 Meningioma,” *World Neurosurg.*, vol. 142, pp. e58–e65, Oct. 2020, doi: 10.1016/J.WNEU.2020.05.145.

[140] F. Maiuri, M. Del Basso De Caro, O. de Divitiis, E. Guadagno, and G. Mariniello, “Recurrence of spinal meningiomas: analysis of the risk factors,” *Br. J. Neurosurg.*, vol. 34, no. 5, pp. 569–574, 2020, doi: 10.1080/02688697.2019.1638886.

[141] P. Sievers *et al.*, “CDKN2A/B homozygous deletion is associated with early recurrence in meningiomas,” *Acta Neuropathol.*, vol. 140, no. 3, pp. 409–413, Sep. 2020, doi: 10.1007/S00401-020-02188-W.

[142] B. E. Lippitz, J. Bartek, T. Mathiesen, and P. Förander, “Ten-year follow-up after Gamma Knife radiosurgery of meningioma and review of the literature,” *Acta Neurochir. (Wien).*, vol. 162, no. 9, pp. 2183–2196, Sep. 2020, doi: 10.1007/S00701-020-04350-5.

[143] Y. Y. Zhang, L. Zhang, Y. Z. Liu, R. Zhang, and G. H. Zhang, “Prognostic factors and long-term outcomes of primary intracranial rhabdoid meningioma: A systematic review,” *Clin. Neurol. Neurosurg.*, vol. 196, Sep. 2020, doi: 10.1016/J.CLINEURO.2020.105971.

[144] P. Soni *et al.*, “Clear Cell Meningioma: A Clinicopathologic Study of a Rare Meningioma Subtype in 35 Patients,” *World Neurosurg.*, vol. 141, pp. e334–e340, Sep. 2020, doi: 10.1016/J.WNEU.2020.05.120.

[145] S. Karimi *et al.*, “Programmed death ligand-1 (PD-L1) expression in meningioma; prognostic significance and its association with hypoxia and NFKB2 expression,” *Sci. Rep.*, vol. 10, no. 1, Dec. 2020, doi: 10.1038/S41598-020-70514-Z.

[146] C. B. de Oliveira Silva *et al.*, “DNA repair and cell synthesis proteins: immunohistochemical expression and correlation with recurrence-regrowth in meningiomas,” *J. Mol. Histol.*, vol. 51, no. 4, pp. 411–420, Aug. 2020, doi: 10.1007/S10735-020-09892-7.

[147] H. J. Oh *et al.*, “Hypofractionated stereotactic radiosurgery for large-sized skull base meningiomas,” *J. Neurooncol.*, vol. 149, no. 1, pp. 87–93, Aug. 2020, doi: 10.1007/S11060-020-03575-9.

[148] H. I. Assi *et al.*, “Demographics and outcomes of meningioma patients treated at a tertiary care center in the Middle East,” *Clin. Neurol. Neurosurg.*, vol. 195, Aug. 2020, doi: 10.1016/J.CLINEURO.2020.105846.

[149] F. Maiuri *et al.*, “Multicentric and diffuse recurrences of meningiomas,” *Br. J. Neurosurg.*, vol. 34, no. 4, pp. 439–446, 2020, doi: 10.1080/02688697.2020.1754335.

[150] D. C. Spille *et al.*, “Risk of tumor recurrence in intracranial meningiomas: comparative analyses of the predictive value of the postoperative tumor volume and the Simpson classification,” *J. Neurosurg.*, vol. 134, no. 6, pp. 1764–1771, Jun. 2020, doi: 10.3171/2020.4.JNS20412.

[151] M. V. Corniola, J. M. Lemée, and T. R. Meling, “Histological transformation in recurrent WHO grade I meningiomas,” *Sci. Rep.*, vol. 10, no. 1, Dec. 2020, doi: 10.1038/S41598-020-68177-X.

[152] P. Pou *et al.*, “Long-Term Outcomes After Linac Radiosurgery for Benign Meningiomas,” *Clin. Oncol. (R. Coll. Radiol).*, vol. 32, no. 7, pp. 452–458, Jul. 2020, doi: 10.1016/J.CLON.2020.02.006.

[153] M. Kim, Y. H. Cho, J. H. Kim, C. J. Kim, S. W. Roh, and D. H. Kwon, “Role of gamma knife radiosurgery for recurrent or residual World Health Organization grade II and III intracranial meningiomas,” *Br. J. Neurosurg.*, vol. 34, no. 3, pp. 239–245, May 2020, doi: 10.1080/02688697.2020.1726285.

[154] A. Fioravanzo *et al.*, “A Risk Score Based on 5 Clinico-Pathological Variables Predicts Recurrence of Atypical Meningiomas,” *J. Neuropathol. Exp. Neurol.*, vol. 79, no. 5, pp. 500–507, May 2020, doi: 10.1093/JNEN/NLAA018.

[155] Y. J. Hu, Y. B. Xie, L. F. Zhang, C. Ding, and J. Chen, “Comparison of clinical outcomes in patients who underwent Gamma Knife radiosurgery for parasellar meningiomas with or without prior surgery,” *BMC Neurol.*, vol. 20, no. 1, Apr. 2020, doi: 10.1186/S12883-020-01731-2.

[156] L. Hua *et al.*, “Long-term outcomes of multimodality management for parasagittal meningiomas,” *J. Neurooncol.*, vol. 147, no. 2, pp. 441–450, Apr. 2020, doi: 10.1007/S11060-020-03440-9.

[157] C. Mirian *et al.*, “Poor prognosis associated with TERT gene alterations in meningioma is independent of the WHO classification: an individual patient data meta-analysis,” *J. Neurol. Neurosurg. Psychiatry*, vol. 91, no. 4, pp. 378–387, Apr. 2020, doi: 10.1136/JNNP-2019-322257.

[158] J. Ma *et al.*, “Low Expression of Phosphatase and Tensin Homolog and High Expression of Ki-67 as Risk Factors of Prognosis in Cranial Meningiomas,” *World Neurosurg.*, vol. 136, pp. e196–e203, Apr. 2020, doi: 10.1016/J.WNEU.2019.12.108.

[159] J. M. Lemée *et al.*, “WHO grade I meningiomas: classification-tree for prognostic factors of survival,” *Neurosurg. Rev.*, vol. 43, no. 2, pp. 749–758, Apr. 2020, doi: 10.1007/S10143-019-01117-0.

[160] A. D. Maier *et al.*, “Clinical and histopathological predictors of outcome in malignant meningioma,” *Neurosurg. Rev.*, vol. 43, no. 2, pp. 643–653, Apr. 2020, doi: 10.1007/S10143-019-01093-5.

[161] Y. Jiang *et al.*, “Clinical features, radiological findings, and treatment outcomes of high-grade lateral ventricular meningiomas: a report of 26 cases,” *Neurosurg. Rev.*, vol. 43, no. 2, pp. 565–573, Apr. 2020, doi: 10.1007/S10143-019-01078-4.

[162] S. Nakasu and Y. Nakasu, “Natural History of Meningiomas: Review with Meta-analyses,” *Neurol. Med. Chir. (Tokyo).*, vol. 60, no. 3, pp. 109–120, 2020, doi: 10.2176/NMC.RA.2019-0213.

[163] C. L. Rogers *et al.*, “High-risk Meningioma: Initial Outcomes From NRG Oncology/RTOG 0539,” *Int. J. Radiat. Oncol. Biol. Phys.*, vol. 106, no. 4, pp. 790–799, Mar. 2020, doi: 10.1016/J.IJROBP.2019.11.028.

[164] Z. Zhao *et al.*, “Treatment Strategy for Petroclival Meningiomas Based on a Proposed Classification in a Study of 168 Cases,” *Sci. Rep.*, vol. 10, no. 1, Dec. 2020, doi: 10.1038/S41598-020-61497-Y.

[165] C. L. Dalle Ore *et al.*, “Hyperostosing sphenoid wing meningiomas: surgical outcomes and strategy for bone resection and multidisciplinary orbital reconstruction,” *J. Neurosurg.*, vol. 134, no. 3, pp. 711–720, Mar. 2020, doi: 10.3171/2019.12.JNS192543.

[166] K. Nakazaki, K. Hara, M. Nishigaki, and M. Uno, “Evaluation of radiological recurrence patterns following gamma knife radiosurgery for solitary meningioma previously treated via cranial surgery,” *J. Clin. Neurosci.*, vol. 73, pp. 24–30, Mar. 2020, doi: 10.1016/J.JOCN.2020.02.019.

[167] J. Deng *et al.*, “Expression and Clinical Significance of Immune Checkpoint Regulator B7-H3 (CD276) in Human Meningioma,” *World Neurosurg.*, vol. 135, pp. e12–e18, Mar. 2020, doi: 10.1016/J.WNEU.2019.10.044.

[168] A. I. Islim *et al.*, “A prognostic model to personalize monitoring regimes for patients with incidental asymptomatic meningiomas,” *Neuro. Oncol.*, vol. 22, no. 2, pp. 278–289, Feb. 2020, doi: 10.1093/NEUONC/NOZ160.

[169] N. Liu, S. Y. Song, J. B. Jiang, T. J. Wang, and C. X. Yan, “The prognostic role of Ki-67/MIB-1 in meningioma: A systematic review with meta-analysis,” *Medicine (Baltimore).*, vol. 99, no. 9, 2020, doi: 10.1097/MD.0000000000018644.

[170] T. Graillon *et al.*, “Everolimus and Octreotide for Patients with Recurrent Meningioma: Results from the Phase II CEVOREM Trial,” *Clin. Cancer Res.*, vol. 26, no. 3, pp. 552–557, Feb. 2020, doi: 10.1158/1078-0432.CCR-19-2109.

[171] H. Zhang, L. Ma, C. Shu, L. qiang Dong, Y. qun Ma, and Y. Zhou, “Spinal Clear Cell Meningiomas: Clinical Features and Factors Predicting Recurrence,” *World Neurosurg.*, vol. 134, pp. e1062–e1076, Feb. 2020, doi: 10.1016/J.WNEU.2019.11.093.

[172] H. Kim *et al.*, “Forkhead box M1 (FOXM1) transcription factor is a key oncogenic driver of aggressive human meningioma progression,” *Neuropathol. Appl. Neurobiol.*, vol. 46, no. 2, pp. 125–141, Feb. 2020, doi: 10.1111/NAN.12571.

[173] Y. M. Gozal, G. Alzhrani, H. Abou-Al-Shaar, M. A. Azab, M. T. Walsh, and W. T. Couldwell, “Outcomes of decompressive surgery for cavernous sinus meningiomas: long-term follow-up in 50 patients,” *J. Neurosurg.*, vol. 132, no. 2, pp. 380–387, 2020, doi: 10.3171/2018.10.JNS181480.

[174] A. Talacchi *et al.*, “Long-term follow-up after surgical removal of meningioma of the inner third of the sphenoidal wing: outcome determinants and different strategies,” *Neurosurg. Rev.*, vol. 43, no. 1, pp. 109–117, Feb. 2020, doi: 10.1007/S10143-018-1018-1.

[175] S. Samal, A. Patnaik, F. M. Sahu, and S. Purkait, “Altered expression of epigenetic modifiers EZH2, H3K27me3, and DNA methyltransferases in meningiomas - prognostic biomarkers for routine practice,” *Folia Neuropathol.*, vol. 58, no. 2, pp. 133–142, Jun. 2020, doi: 10.5114/FN.2020.96970.

[176] P. Karabagli, H. Karabagli, Z. Mavi, F. Demir, and E. Y. Ozkeles, “Histopathological and Clinical Features as Prognostic Factors of Atypical Meningiomas,” *Turk. Neurosurg.*, vol. 30, no. 5, pp. 746–757, 2020, doi: 10.5137/1019-5149.JTN.31161-20.1.

[177] N. Keric *et al.*, “Impact of postoperative radiotherapy on recurrence of primary intracranial atypical meningiomas,” *J. Neurooncol.*, vol. 146, no. 2, pp. 347–355, Jan. 2020, doi: 10.1007/S11060-019-03382-X.

[178] M. A. Mooney *et al.*, “Brachytherapy with surgical resection as salvage treatment for recurrent high-grade meningiomas: a matched cohort study,” *J. Neurooncol.*, vol. 146, no. 1, pp. 111–120, Jan. 2020, doi: 10.1007/S11060-019-03342-5.

[179] N. Lamba *et al.*, “Atypical Histopathological Features and the Risk of Treatment Failure in Nonmalignant Meningiomas: A Multi-Institutional Analysis,” *World Neurosurg.*, vol. 133, pp. e804–e812, Jan. 2020, doi: 10.1016/J.WNEU.2019.10.002.
