# Supplementary material for: The importance of considering competing risks in recurrence analysis of intracranial meningioma
Source: J Neurooncol. 2024 Feb 10;166(3):503–11. doi: 10.1007/s11060-024-04572-y (PMC10876814; doi:10.1007/s11060-024-04572-y)
Supplement: Supplementary file 2 — Supplementary file2 (DOCX 21 KB) [file 11060_2024_4572_MOESM2_ESM.docx]

| **age_group** | **WHO** | **1 - Kaplan (2.5yrs)** | **Aalen Johansen (2.5 yrs)** | **1 - Kaplan (5 yrs)** | **Aalen Johansen (5 yrs)** | **1 - Kaplan (7.5 yrs)** | **Aalen Johansen (7.5 yrs)** | **1 - Kaplan (10 yrs)** | **Aalen Johansen (10 yrs)** |
| --- | --- | --- | --- | --- | --- | --- | --- | --- | --- |
| <60 | WHO1 | 6.3% (5.2 to 7.4) | 6.3% (5.2 to 7.4) | 13.1% (11.4 to 14.8) | 13.0% (11.3 to 14.7) | 17.7% (15.6 to 19.8) | 17.5% (15.5 to 19.6) | 21.0% (18.5 to 23.5) | 20.7% (18.3 to 23.2) |
| <60 | WHO2 | 14.8% (12.0 to 17.6) | 14.7% (12.0 to 17.5) | 26.7% (22.8 to 30.7) | 26.5% (22.6 to 30.4) | 34.5% (29.9 to 39.2) | 34.1% (29.5 to 38.7) | 39.5% (34.1 to 44.8) | 38.9% (33.7 to 44.2) |
| <60 | WHO3 | 35.3% (19.8 to 50.8) | 32.1% (17.7 to 46.6) | 56.1% (39.5 to 72.6) | 50.7% (35.0 to 66.4) | 56.1% (39.5 to 72.6) | 50.7% (35.0 to 66.4) | 73.6% (52.3 to 94.9) | 65.6% (45.9 to 85.2) |
| 60-69 | WHO1 | 4.8% (3.5 to 6.2) | 4.8% (3.4 to 6.1) | 9.8% (7.7 to 12.0) | 9.6% (7.5 to 11.6) | 15.7% (12.6 to 18.7) | 15.1% (12.2 to 18.1) | 19.9% (16.1 to 23.7) | 19.0% (15.4 to 22.6) |
| 60-69 | WHO2 | 17.2% (13.1 to 21.2) | 16.8% (12.8 to 20.7) | 30.6% (25.3 to 35.9) | 29.6% (24.4 to 34.7) | 39.6% (33.5 to 45.8) | 37.8% (32.0 to 43.7) | 44.7% (38.0 to 51.4) | 42.3% (36.0 to 48.6) |
| 60-69 | WHO3 | 56.3% (35.1 to 77.6) | 50.0% (30.0 to 70.0) | 62.5% (41.1 to 84.0) | 54.7% (34.6 to 74.8) | 90.6% (74.7 to 100.0) | 75.9% (57.4 to 94.3) | NA (NA to NA) | NA (NA to NA) |
| 70-79 | WHO1 | 6.6% (4.5 to 8.6) | 6.4% (4.4 to 8.4) | 9.9% (7.2 to 12.6) | 9.6% (7.0 to 12.1) | 12.9% (9.6 to 16.2) | 12.2% (9.1 to 15.3) | 18.0% (13.2 to 22.9) | 16.0% (11.9 to 20.0) |
| 70-79 | WHO2 | 23.4% (17.6 to 29.2) | 22.2% (16.7 to 27.7) | 34.3% (27.4 to 41.2) | 32.0% (25.5 to 38.5) | 38.3% (30.7 to 45.9) | 35.3% (28.3 to 42.3) | 40.8% (32.1 to 49.5) | 36.9% (29.4 to 44.3) |
| 70-79 | WHO3 | 65.0% (40.9 to 89.1) | 55.0% (33.2 to 76.8) | NA (NA to NA) | NA (NA to NA) | NA (NA to NA) | NA (NA to NA) | NA (NA to NA) | NA (NA to NA) |
| >80 | WHO1 | 6.3% (2.0 to 10.6) | 5.8% (1.8 to 9.7) | 8.5% (3.3 to 13.8) | 7.8% (3.0 to 12.5) | 8.5% (3.3 to 13.8) | 7.8% (3.0 to 12.5) | 12.2% (3.6 to 20.8) | 10.0% (3.7 to 16.3) |
| >80 | WHO2 | 22.4% (10.4 to 34.3) | 20.3% (9.5 to 31.2) | 31.0% (16.9 to 45.1) | 27.5% (15.0 to 40.0) | 47.7% (24.1 to 71.4) | 35.8% (20.4 to 51.1) | 47.7% (24.1 to 71.4) | 35.8% (20.4 to 51.1) |
| >80 | WHO3 | 75.0% (32.6 to 100.0) | 60.0% (17.1 to 100.0) | 75.0% (32.6 to 100.0) | 60.0% (17.1 to 100.0) | 75.0% (32.6 to 100.0) | 60.0% (17.1 to 100.0) | 75.0% (32.6 to 100.0) | 60.0% (17.1 to 100.0) |

**Supplementary Table 2**

Results obtained with the two methods: Aalen-Johansen and Kaplan-Meier, at time points 2.5, 5.0, 7.5, and 10 years in relation to age and WHO grade.

**The importance of considering competing risks in recurrence analysis of intracranial meningioma**

Christian Mirian^1 (ORC ID: 0000-0001-6801-0123)^, Lasse Rehné Jensen^1 (ORC ID: 0000-0001-6931-4399)^ , Tareq A. Juratli^2,3 (ORC ID: 0000-0003-2236-6719)^, Andrea Daniela Maier^1,4 (ORC ID: 0000-0002-5930-0636)^, Sverre H. Torp^5,6^, Helen A. Shih^7^, Ramin A. Morshed^8^, Jacob S. Young^8 (ORC ID: 0000-0002-5499-4325)^, Stephen T. Magill^8,9 (ORC ID: 0000-0002-5257-7835)^, Luca Bertero^10 (ORC ID: 0000-0001-9887-7668)^, Walter Stummer^11^, Dorothee Cäcilia Spille^11^, Benjamin Brokinkel^11, 12^, Soichi Oya^13^, Satoru Miyawaki^14^, Nobuhito Saito^14^, Martin Proescholdt^15^, Yasuhiro Kuroi^16^, Konstantinos Gousias^17^, Matthias Simon^18^, Jennifer Moliterno^19^, Ricardo Prat-Acin^20^, Stéphane Goutagny^21^, Vikram C. Prabhu^22^, John T. Tsiang^22 (ORC ID: 0000-0001-5869-6408)^, Johannes Wach^23^, Erdem Güresir^23^, Junkoh Yamamoto^24^, Young Zoon Kim^25 (ORC ID: 0000-0003-1171-0780)^, Joo Ho Lee^26^, Matthew Koshy^27^, Karthikeyan Perumal^28^, Mustafa K. Baskaya^28^, Donald M. Cannon^29^, Dennis C. Shrieve^29^, Chang-Ok Suh^30^, Jong Hee Chang^31^, Maria Kamenova^32^, Sven Straumann^32^, Jehuda Soleman^32^, Ilker Y. Eyüpoglu^2^, Tony Catalan^8 (ORC ID: 0009-0005-8821-3880)^, Austin Lui^8 (ORC ID: 0000-0003-1347-8815)^, Philip V. Theodosopoulos^8^, Michael W. McDermott^8,33^, Fang Wang^34^, Fuyou Guo^34^, Pedro Góes^35^, Manoel Antonio de Paiva Neto^35^, Aria Jamshidi^36^, Ricardo Komotar^36^ , Michael Ivan^36^, Evan Luther^36^, Luis Souhami^37^, Marie-Christine Guiot^38^, Tamás Csonka^39^, Toshiki Endo^40^, Olivia Claire Barrett^41^, Randy Jensen^42^, Tejpal Gupta^43^, Akash J. Patel^44,45,46^, Tiemo J. Klisch^46,47^, Jun Won Kim^48^, Francesco Maiuri^49^, Valeria Barresi^50^, María Dolores Tabernero^51 (ORC ID: 0000-0002-4430-9806)^, Simon Skyrman^52^, Anders B. Jørgensen^1^, Mathias Jacobsen Bach^1^, Ian Law^53,54^, David Scheie^4^, Bjarne Winther Kristensen^4,55^, Tina Nørgaard Munch^1,54,56^, Torstein Meling^1,57^, Kåre Fugleholm^1,54^, Paul Blanche^58^, Tiit Mathiesen^1,52,54^

**Corresponding author**

Christian Mirian, MD

[Christian.mirian.larsen@regionh.dk](mailto:Christian.mirian.larsen@regionh.dk)

**Affiliations**

1: Department of Neurosurgery, Copenhagen University Hospital, Copenhagen, Denmark

2: Department of Neurosurgery, Division of Neuro-Oncology, Faculty of Medicine and University Hospital Carl Gustav Carus, Technische Universität Dresden, 01307 Dresden, Germany

3: Department of Neurosurgery, Laboratory of Translational Neuro-Oncology, Massachusetts General Hospital Cancer Center, Harvard Medical School, Boston, USA

4: Department of Pathology, Bartholin Institute, Rigshospitalet, Copenhagen University Hospital, Copenhagen, Denmark

5: Department of Clinical and Molecular Medicine, Faculty of Medicine and Health Sciences, Norwegian, University of Science and Technology (NTNU), Laboratory Centre, St. Olavs hospital, NO-7491 Trondheim, Norway,

6: Department of Pathology, Laboratory Centre, St. Olavs hospital, NO-7030 Trondheim, Norway

7: Department of Radiation Oncology, Massachusetts General Hospital, Harvard Medical School, Boston, MA, USA

8: Department of Neurological Surgery, University of California San Francisco, San Francisco, California, USA

9: Department of Neurological Surgery, Northwestern University, Feinberg School of Medicine, Illinois, USA

10: Pathology Unit, Department of Medical Sciences, University and Città della Salute e della Scienza University Hospital of Turin, Turin, Italy

11: Department of Neurosurgery, University of Münster, Münster, Germany

12: Institute for Neuropathology, University of Münster, Münster, Germany

13: Department of Neurosurgery, Saitama Medical Center/University, Saitama, Japan

14: Department of Neurosurgery, The University of Tokyo Hospital, Tokyo, Japan

15: Department of Neurosurgery, University Regensburg Medical Center, Regensburg, Germany

16: Department of Neurosurgery, Tokyo Women's Medical University, Adachi Medical Center, Tokyo, Japan

17: Department of Neurosurgery, Athens Medical Center, Athens, Greece

18: Department of Neurosurgery, Bethel Clinic University of Bielefeld Medical Center, Bielefeld, Germany

19: Department of Neurosurgery, Yale School of Medicine Yale New Haven Hospital, Smilow Cancer Hospital, New Haven, USA

20: Department of Neurosurgery, Hospital La Fe, Valencia. Spain

21: Université Paris Cité, Department of Neurosurgery, Beaujon Hospital, Assistance Publique Hôpitaux de Paris, Paris, France

22: Department of Neurological Surgery, Loyola University Medical Center, Stritch School of Medicine, Illinois, USA

23: Department of Neurosurgery, University Hospital Leipzig, Leipzig, Germany

24: Department of Neurosurgery, University of Occupational and Environmental Health, Kitakyushu, Japan

25: Department of Neurosurgery, Samsung Changwon Hospital, Sungkyunkwan University School of Medicine, Changwon, Republic of Korea

26: Department of Radiation Oncology, Seoul National University Hospital, Seoul National University College of Medicine, Seoul, Republic of Korea.

27: Department of Radiation Oncology, University of Illinois Hospital and Health Sciences System, Illinois, USA

28: Department of Neurosurgery, University of Wisconsin Medical School & Public Health, Madison, Wisconsin, USA

29: Department of Radiation Oncology Spencer Fox Eccles School of Medicine University of Utah, Utah, USA

30: Department of Radiation Oncology, Yonsei University College of Medicine, Seoul, Republic of Korea

31: Department of Neurosurgery, Yonsei University College of Medicine, Seoul, Republic of Korea

32: Department of Neurosurgery, University Hospital Basel, Basel, Switzerland.

33: Division of Neurosurgery, Miami Neuroscience Institute, Miami, Florida, USA

34: Department of Neurosurgery, The First Affiliated Hospital of Zhengzhou University, Zhengzhou, Henan, China

35: Department of Neurosurgery, Federal University of São Paulo, São Paulo, Brazil

36 Department of Neurological Surgery, Sylvester Comprehensive Cancer Center, University of Miami, Florida, USA

37: Division of Radiation Oncology, McGill University Health Centre, McGill University, Montreal, Quebec, Canada

38: Department of Pathology, McGill University Health Centre, Montreal, Quebec, Canada

39: Department of Pathology, Faculty of Medicine, University of Debrecen, Hungary

40: Division of Neurosurgery, Tohoku Medical and Pharmaceutical University, Tohoku, Japan

41: Infirmary Cancer Care, Alabama, USA

42: Department of Neurosurgery, Huntsman Cancer Institute, University of Utah, Salt Lake City, Utah, USA

43: Department of Radiation Oncology ACTREC, Tata Memorial Centre, HBNI Kharghar, Navi Mumbai:410210, India

44: Department of Neurosurgery, Baylor College of Medicine, Houston, Texas, USA

45: Department of Otolaryngology-Head and Neck Surgery, Baylor College of Medicine, Houston, TX

46: Jan and Dan Duncan Neurological Research Institute, Texas Children’s Hospital, Houston, TX

47: Department of Molecular and Human Genetics, Baylor College of Medicine, Houston, TX, USA.

48: Department of Radiation Oncology, Gangnam Severance Hospital, Yonsei University College of Medicine, Seoul, Republic of Korea

49: Department of Neurosurgery, University of Naples Federico II, Naples, Italy

50: Department of Diagnostics and Public Health, University of Verona, Italy

51: Instituto de Investigación Biomédica de Salamanca (IBSAL), University Hospital of Salamanca, Salamanca, Spain

52: Department of Clinical Neuroscience, Karolinska Institutet, Stockholm, Sweden

53: Department of Clinical Physiology and Nuclear Medicine, Copenhagen University Hospital-Rigshospitalet, Copenhagen, Denmark

54: Department of Clinical Medicine, Faculty of Health and Medical Sciences, University of Copenhagen, Copenhagen, Denmark.

55: Department of Clinical Medicine and Biotech Research and Innovation Center (BRIC), University of Copenhagen, Copenhagen, Denmark

56: Department of Epidemiology Research, Statens Serum Institut, Copenhagen, Denmark

57: Department of Neurological Surgery, Istituto Nazionale Neurologico "C.Besta", Milan, Italy

58: Section of Biostatistics, Department of Public Health, University of Copenhagen, Copenhagen, Denmark.
